# High rate of return to low‐impact physical activity or sports after total and unicompartmental knee arthroplasty: A systematic review with meta‐analysis

**DOI:** 10.1002/ksa.70267

**Published:** 2026-01-13

**Authors:** Rodrigo Braz, Eluana Gomes, Cristina Valente, Hamada Ahmed, Mohamed Moursy, Diego Delgado, Mikel Sanchez, Renato Andrade, João Espregueira‐Mendes

**Affiliations:** ^1^ School of Medicine University of Minho Braga Portugal; ^2^ Clínica Espregueira ‐ FIFA Medical Centre of Excellence Porto Portugal; ^3^ Dom Henrique Research Centre Porto Portugal; ^4^ Physical Medicine & Rehabilitation, Medical Affairs Department Sultan Bin Abdulaziz Humanitarian City Riyadh Saudi Arabia; ^5^ Advanced Biological Therapy Unit, MiKS Hospital Vitoria‐Gasteiz Spain; ^6^ Arthroscopic Surgery Unit, MiKS Hospital Vitoria‐Gasteiz Spain; ^7^ Porto Biomechanics Laboratory (LABIOMEP), Faculty of Sports University of Porto Porto Portugal; ^8^ ICVS/3B′s‐PT Government Associate Laboratory Braga Portugal; ^9^ 3B′s Research Group—Biomaterials, Biodegradables and Biomimetics University of Minho, Headquarters of the European Institute of Excellence on Tissue Engineering and Regenerative Medicine Barco Portugal

**Keywords:** arthroplasty, knee, physical activity, sports

## Abstract

**Purpose:**

Rates and time to return to physical activity (PA) or sports following knee arthroplasty remain poorly explored in the current literature. This systematic review with meta‐analysis aimed to synthesise the return to PA or sports after knee arthroplasty, comparing between total knee arthroplasty (TKA) and unicompartmental knee arthroplasty (UKA).

**Methods:**

PubMed, Web of Science and Embase databases were searched up to December 2025 to identify studies reporting the return to PA or sports following knee arthroplasty. A meta‐analysis was performed to evaluate the outcomes and to compare results between TKA and UKA. The rate of return to PA or sports was reported as pooled proportions, while comparisons between TKA and UKA for dichotomous outcomes were expressed as odds ratios (ORs) with corresponding 95% confidence intervals (CIs).

**Results:**

Forty‐five studies were included, encompassing 14,498 knees from 13,716 individuals (65.5 ± 3.5 years) who underwent knee arthroplasty. The overall rate of return to PA or sports was high (85.2%, 95% CI: 76.8%–92.1%), within a median of 4.1 months. The likelyhood of returning to PA or sports was significantly lower after TKA than UKA (OR = 0.31, 95% CI: 0.12–0.82). Certainty of evidence across all outcomes was rated as very low. Patients displayed a postoperative increase in PA scores was observed, and reported satisfaction rates ranged from 61% to 100%. Participation in high‐impact sports declined, with the most commonly cited reason being precautionary behaviour.

**Conclusions:**

Most patients can expect to resume PA or sports within a short timeframe after knee arthroplasty, particularly to low‐impact activities. Those with UKA displayed higher likelihood to return to PA or sports. Participation in high‐impact sports often declines, largely due to psychological factors that may limit participation in PA beyond the actual physical capabilities, underscoring the need for better patient counselling.

**Level of Evidence:**

Level IV.

**Abbreviations**
CI: confidence interval
GRADE: Grading of Recommendations Assessment, Development and Evaluation
HAAS: high‐activity arthroplasty score
I^2^
: I‐squared
IQR: interquartile range
LEAS: lower extremity activity scale
MD: mean difference
OA: knee osteoarthritis
ORs: odds ratios
PA: physical activity
PICOS: participants intervention comparator outcomes and study
PRISMA: preferred reporting items for systematic reviews and meta‐analyses
PROMs: patient‐reported outcome measures
RoBANS: Risk of Bias Assessment tool for Non‐randomised Studies
ROBINS‐I: Risk Of Bias In Non‐randomised Studies ‐ of Interventions
RTS: return to sports
SD: standard deviation
TKA: total knee arthroplasty
UCLA: University of California, Los Angeles Activity Score
UKA: unicompartmental knee arthroplasty

## INTRODUCTION

Knee arthroplasty is a well‐established surgical treatment for end‐stage knee OA, offering substantial pain relief and functional restoration [16]. Among physically active individuals, a common concern following surgery is the ability to return to physical activity (PA) and, in some cases, to sports participation [50]. Reported return to PA after knee arthroplasty ranges from 34% to 100% [31], highlighting substantial variability. Previous studies suggest that patients are more likely to resume sports following unicompartmental knee arthroplasty (UKA) than total knee arthroplasty (TKA) [59]. Beyond the type of surgery, several other factors influence return to PA, including age, the nature and intensity of the activity, and patients′ motivations and expectations regarding postoperative physical performance. Although many patients do successfully resume PA or sports following knee arthroplasty, the variability in outcomes underscores the importance of individualised assessment to guide recovery and manage expectations effectively.

Despite the widespread use of knee arthroplasty, current knowledge on postoperative outcomes, particularly concerning the resumption of PA or sports, remains limited. Much of the existing literature addresses return to activity based on expert opinion and recommendations issued by orthopaedic surgical societies [41], rather than on high‐quality, evidence‐based research. This lack of guidelines backed by scientific evidence contributes to suboptimal postoperative management, especially for patients who wish to return to sports (RTS). Previous reviews often focus on specific populations or one type of arthroplasty, often do not pool data of return to PA or sports, and are currently outdated as they do not include more recent studies. To address this gap, the present systematic review with meta‐analysis aimed to investigate the return to PA or sports among individuals with knee OA undergoing knee arthroplasty, comparing outcomes between TKA and UKA. The summarised evidence based on the return to PA or sports should help guide the prognosis and expectation following knee arthroplasty. Based on previous research [41, 56, 59], it was hypothesised that most patients would return to low‐level PA, with those with UKA returning to a higher rate than TKA.

## METHODS

This systematic review with meta‐analysis was conducted in accordance with the preferred reporting items for systematic reviews and meta‐analyses (PRISMA) 2020 statement [40] and following the recommendations of the PRISMA‐PERSiST consensus [3]. The protocol was prospectively registered in PROSPERO CRD42024512913 and deviations from the original protocol are detailed in Supplement S1.

### Eligibility criteria

The eligibility criteria were structured according to the PICOS framework: Participants, intervention, comparator, outcomes and study design. No restrictions were applied based on publication date and only studies published in English were included.

### Participants

Eligible studies were required to include adult individuals with either primary or post‐traumatic knee OA, regardless of sex, race, or overall health status. No restrictions were applied regarding the type or intensity level of PA.

### Intervention

Eligible studies were required to evaluate the treatment of knee OA using either UKA or TKA. Studies involving concurrent knee procedures that could influence outcomes were excluded. Similarly, studies focusing on revision knee arthroplasty were not considered. Participants undergoing isolated patellofemoral arthroplasty were also excluded.

### Comparator

Comparator groups were not mandatory for inclusion of the study into the systematic review. However, when available, studies that included comparison groups, specifically UKA versus TKA, were included and analysed accordingly.

### Outcomes

The primary outcomes were the rate and time to return to PA or sports following knee arthroplasty. Secondary outcome was the level of PA or sports participation before and after surgery. Studies needed to report any of the primary or secondary outcomes to be eligible for inclusion.

### Study design

Eligible study designs consisted of any type of clinical trials, from randomised controlled trials, cohort studies, case‐control studies, cross‐sectional studies and case series. While case series were considered eligible, only those with a minimum of five participants were included to ensure external validity. All other study designs were excluded.

### Search strategy

The search strategy (Supplement S2) was applied to PubMed, EMBASE and Web of Science databases up to April 20, 2024, and updated on December 2, 2025. Manual searches were performed by screening the reference lists of included studies and relevant reviews to identify any potentially eligible studies that may have been missed in the database searches.

### Study selection

All records retrieved from the databases were imported into EndNote, where duplicates were initially removed using the software tools and subsequently verified manually. Two authors (R.B. and R.A.) independently screened all titles and abstracts to identify potentially relevant studies for inclusion. Following this initial screening, full texts of relevant articles were reviewed to confirm their eligibility. Any disagreements between reviewers were resolved through consensus.

### Data collection

All data were collected by the same two authors (R.B. and R.A.). Extracted data included study and patient characteristics, intervention details, and outcomes related to return to PA or sports (Supplement S3).

### Risk of bias

The risk of bias in comparative studies was judged using the Risk Of Bias In Nonrandomised Studies of Interventions (ROBINS‐I) tool following the tool guidelines [51]. The risk of bias of noncomparative studies was judged according to Risk of Bias Assessment tool for Nonrandomised Studies (RoBANS) [27]. Each domain was judged as low risk of bias, high risk of bias or unclear, with judgement details available at Supplement S4. Two authors (R.B. and R.A.) judged the risk of bias at the study‐level, with any disagreements resolved through discussion until consensus was reached.

### Data synthesis

Data were summarised using pooled means and standard deviations (SD) for continuous outcomes, and frequencies with percentages (%) for categorical outcomes. All pooled data were weighted by sample size to eschew overrepresentation of studies with small or large samples. Median and interquartile range (IQR) were estimated exclusively for time to return to PA or sports to account for data skewness.

All meta‐analyses were conducted using the ‘meta,’ ‘metadata’ and ‘metafor’ packages in R Studio (version 2024.04.2+764) software. The meta‐analysis employed a random‐effects model, inverse variance weighting and Sidik–Jonkman estimator. A minimum of three studies was required for each outcome to be included in the meta‐analysis. Statistical heterogeneity was assessed using the *I*‐squared (*I*²) statistic and classified as not important (<50%), moderate (50%–75%) or high (>75%) [20].

Within‐group meta‐analyses were performed to estimate the pre‐to‐post changes in the Tegner Activity Scale, University of California, Los Angeles Activity Score (UCLA) and the high‐activity arthroplasty score (HAAS). These continuous outcomes were pooled as mean differences (MDs) with 95% confidence intervals (CIs).

A meta‐analysis of the proportion of return to PA or sports was conducted with proportions being transformed using the Freeman–Tukey double arcsine transformation [32] and zero events were corrected using continuity correction adding 0.5 in each cell [4]. A between‐group meta‐analysis was also performed to estimate the odds ratio (OR) of return to PA or sports between UKA and TKA.

Sensitivity analyses were conducted by re‐running the meta‐analyses after excluding studies that included nonactive patients at baseline or that central tendency data had to be estimated. We intended to conduct a sensitivity analysis for our primary outcome excluding studies published longer than 20 years, but all studies reporting the primary outcome were published within the last 20 years.

Funnel plots were generated for visual inspection of the risk of publication bias only when ten or more study entries were eligible for meta‐analysis [1].

### Certainty of evidence

Two authors (R.A. and E.G.) judged the certainty of evidence using the Grading of Recommendations Assessment, Development and Evaluation (GRADE) framework [49]. Certainty was downgraded based on concerns related to risk of bias, inconsistency, imprecision, indirectness, or risk of publication bias. However, when a meta‐analysis included fewer than ten studies, risk of publication bias was not considered for downgrading the certainty of evidence [1]. Certainty of evidence was rated as high, moderate, low or very low certainty.

## RESULTS

### Search results and characteristics of the studies

**Figure 1:**
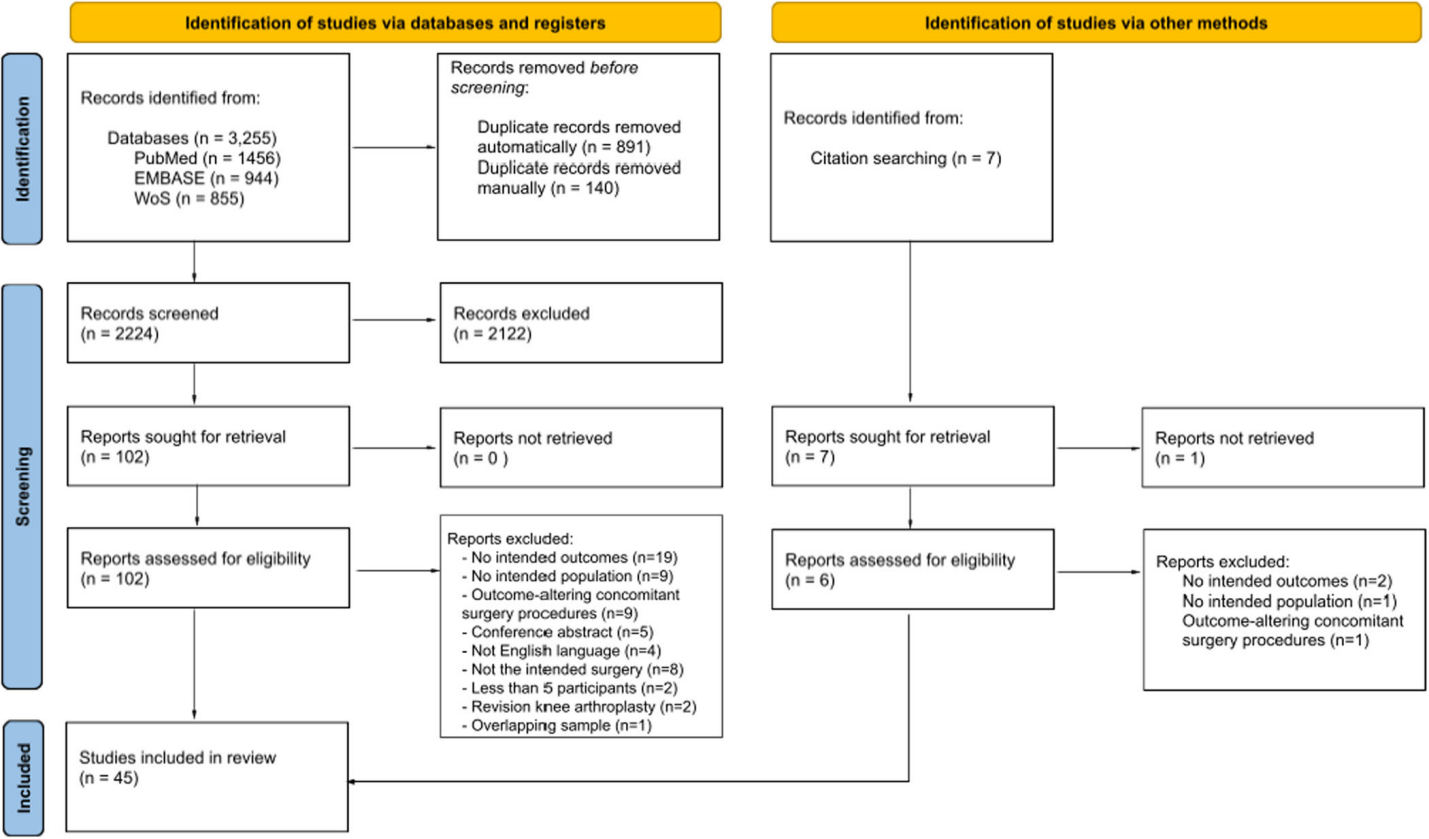
PRISMA flow chart. PRISMA, Preferred Reporting Items for Systematic Reviews and Meta‐Analysis.

Following database and manual searches, a total of 3255 records were identified. After screening, 108 full‐text articles were retrieved and assessed for eligibility. Ultimately, 45 studies [2, 6, 7, 8, 9, 10, 11, 12, 13, 14, 15, 17, 18, 19, 21, 22, 23, 24, 25, 26, 28, 29, 30, 33, 34, 35, 36, 37, 39, 42, 43, 44, 45, 46, 47, 48, 52, 53, 54, 55, 57, 58, 60, 61, 62] met the inclusion criteria and were included in this systematic review (Figure 1).

### Risk of bias and certainty of evidence

**Figure 2:**
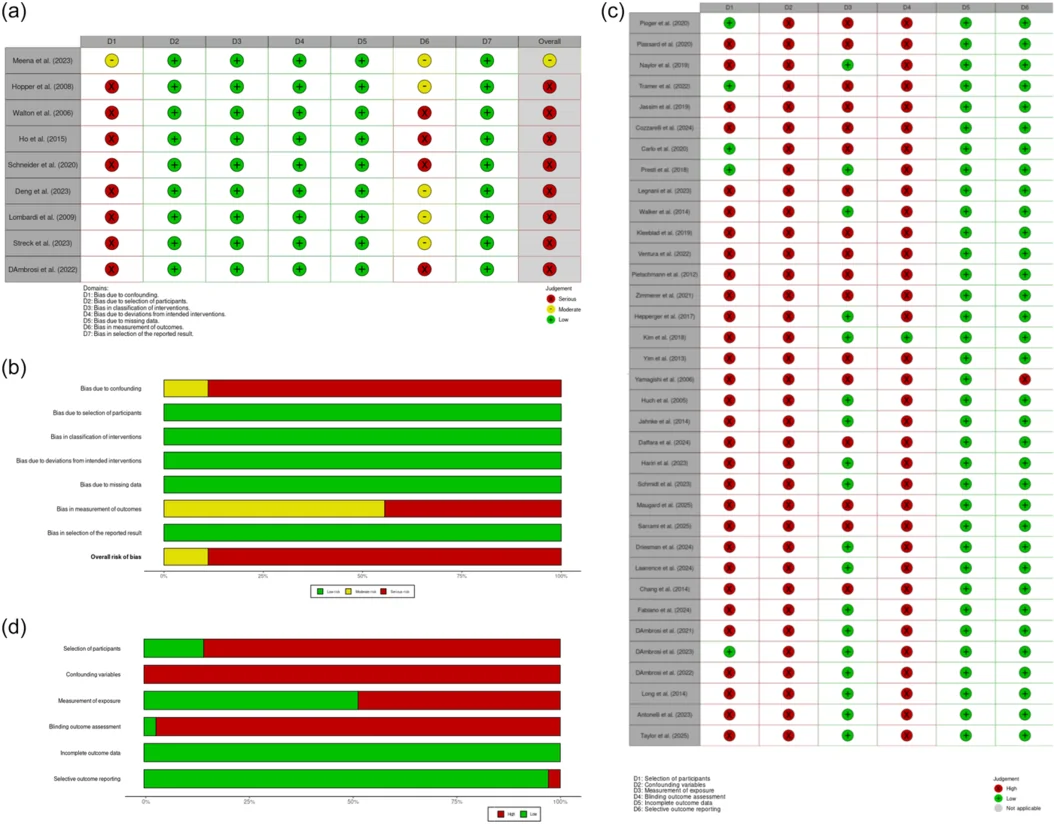
Judgement of risk of bias: Risk Of Bias In Non‐randomised Studies ‐ of Interventions (ROBINS‐I) for non‐randomised comparative studies for each study (a) and summary plot (b); Risk of Bias Assessment tool for Non‐randomised Studies (RoBANS) for noncomparative studies for each study (c) and summary plot (d).

The risk of bias for comparative studies was judged as serious for all but one study, which was judged as moderate risk (Figure 2a). The most concerning bias domains were bias due to confounding with one study with moderate risk and all others with serious risk, and bias due to the measurement of the outcome with five studies with moderate risk and four studies with serious risk. All other domains were judged as low risk of bias (Figure 2b).

The noncomparative studies were judged with high risk of selection bias due to selection of participants (86%), high risk of selection bias due to confounding (100%), high risk of performance bias due to the measurement of outcome (49%) and high risk of detection bias due to missing to blind the outcome evaluators (97%). The remaining two bias domains were judged with low risk of bias (Figure 2c,d).

The certainty of evidence was judged as ‘very low’ for all outcome measures, primarily due to concerns regarding risk of bias and inconsistency (Supplement S5).

### Population characteristics and surgical intervention details

A total of 14,498 knees from 13,716 individuals were included in the analysis, with a mean age of 65.5 ± 3.5 years and 29.2 ± 1.7 kg/m^2^ were included for analysis (Supplement S6). The sample was more skewed towards females (69%) than males (31%). The mean follow‐up duration was 28.0 ± 23.9 months, ranging from 6 months to 25 years.

A greater proportion of patients underwent TKA (79%, *n* = 9360), while 2530 patients received UKA (21%). Detailed information on the arthroplasty procedures can be found in Supplements S7 and S8.

### Return to PA or sports after knee arthroplasty

Most of the included studies reported data on return to PA or sports following knee arthroplasty (Supplement S9). Pre‐operative PA assessments were most commonly described as occurring ‘before surgery’ in 29 studies [2, 6, 7, 9, 10, 11, 12, 13, 14, 15, 17, 19, 22, 23, 24, 25, 30, 34, 35, 36, 43, 44, 45, 52, 54, 55, 58, 60, 61], while three studies [29, 46, 53] assessed PA 12–24 months before surgery and seven studies [18, 21, 26, 33, 47, 57, 62] ‘before the onset of knee symptoms’. Patients were categorised as either physically active (engaging in PA or sports) or nonactive. Overall, the number of physically active patients decreased from 4416 at baseline to 3909 at follow‐up, representing a 11.5% decline. There was a similar decline in active patients post‐operatively for the TKA (19.6% decline, *n* = 639) and UKA (14.4% decline, *n* = 182) subgroups.

**Figure 3:**
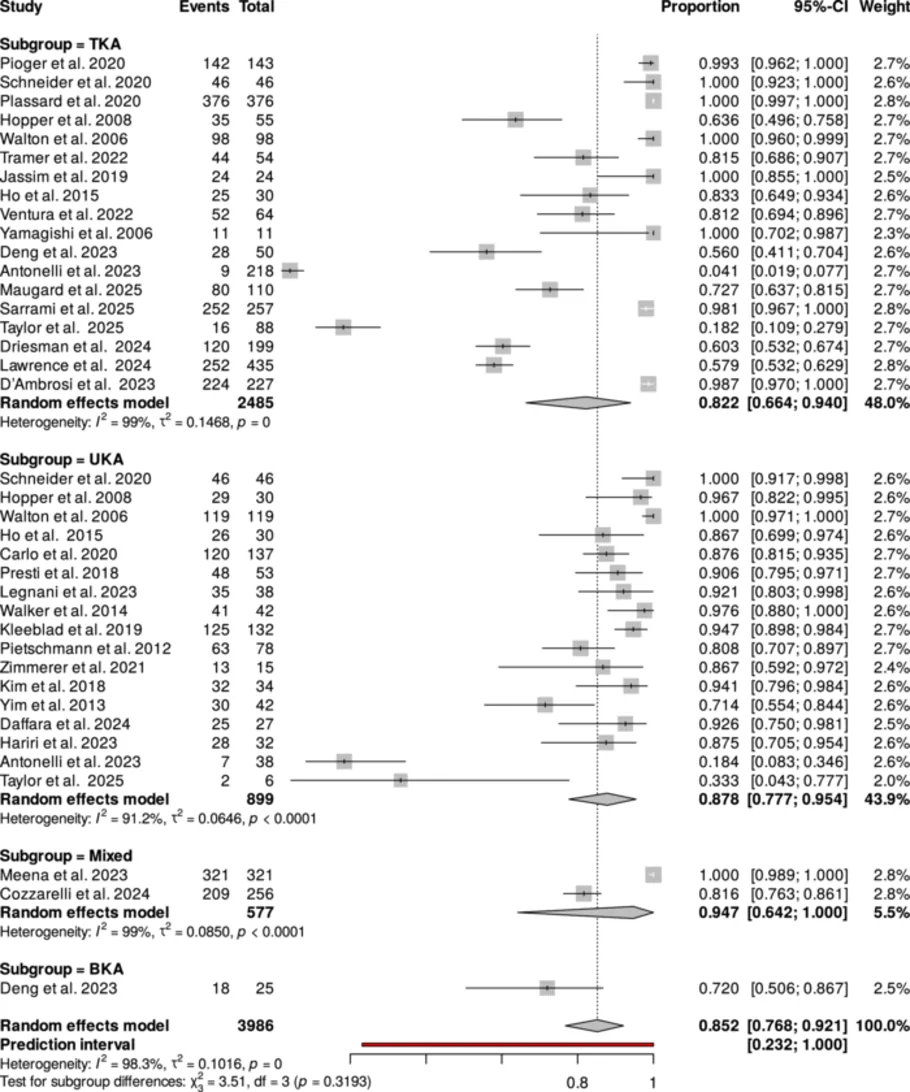
Forrest plot for proportion of return to physical activity (PA) or sports subgrouped by type of knee arthroplasty. CI, confidence interval; TKA, total knee arthroplasty; UKA, unicompartmental knee arthroplasty.

The overall pooled proportion of all patients who returned to PA or sports was 85.2% (95% CI: 76.8%–92.1%; *I*² = 98%; Figure 3). There were no significant differences between the TKA and UKA subgroups, with UKA showing a pooled proportion of return to PA or sports of 87.8% (95% CI: 77.7%–95.4%; *I*² = 91%) and TKA of 82.2% (95% CI: 66.4%–94.0%; *I*² = 83%). Sensitivity analyses did not change the pooled estimates or the degree of heterogeneity (Supplements S10). The funnel plot was asymmetric, suggesting risk of publication bias (Supplement S11).

**Figure 4:**
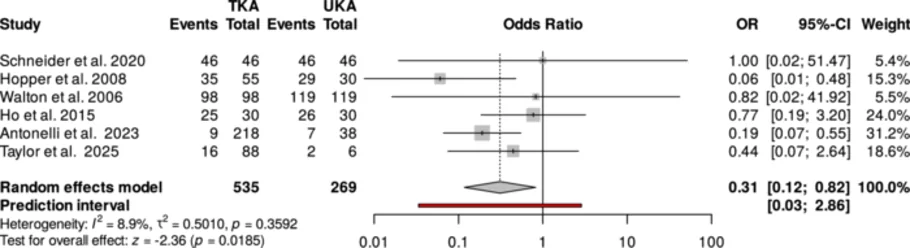
Forrest Plot for comparison of return to PA or sports proportion between TKA and UKA. CI, confidence interval; PA, physical activity; TKA, total knee arthroplasty; UKA, unicompartmental knee arthroplasty.

The between‐group meta‐analysis of comparative studies revealed that those with TKA displayed a lower likelihood of returning to PA or sports than UKA (OR = 0.31, 95% CI: 0.12–0.82; *I*² = 9%; Figure 4). Sensitivity analyses did not change the pooled estimates or the degree of heterogeneity (Supplements S12). Only one study [48] provided data on RTS stratified by level of impact and performance, showing a higher proportion of patients in the UKA group returning to similar or higher impact sports compared to the TKA group (76.1% vs. 54.1%).

Eleven studies [6, 21, 22, 26, 28, 34, 36, 44, 45, 47, 54] reported the time to return to PA or sports, resulting in a pooled median of 4.1 months (IQR: 3.7–5.2). The median time was comparable between the TKA subgroup (4.1 months, IQR: 3.9–5.2) and the UKA subgroup (3.8 months, IQR: 3.2–5.0).

### PA scores

**Figure 5:**
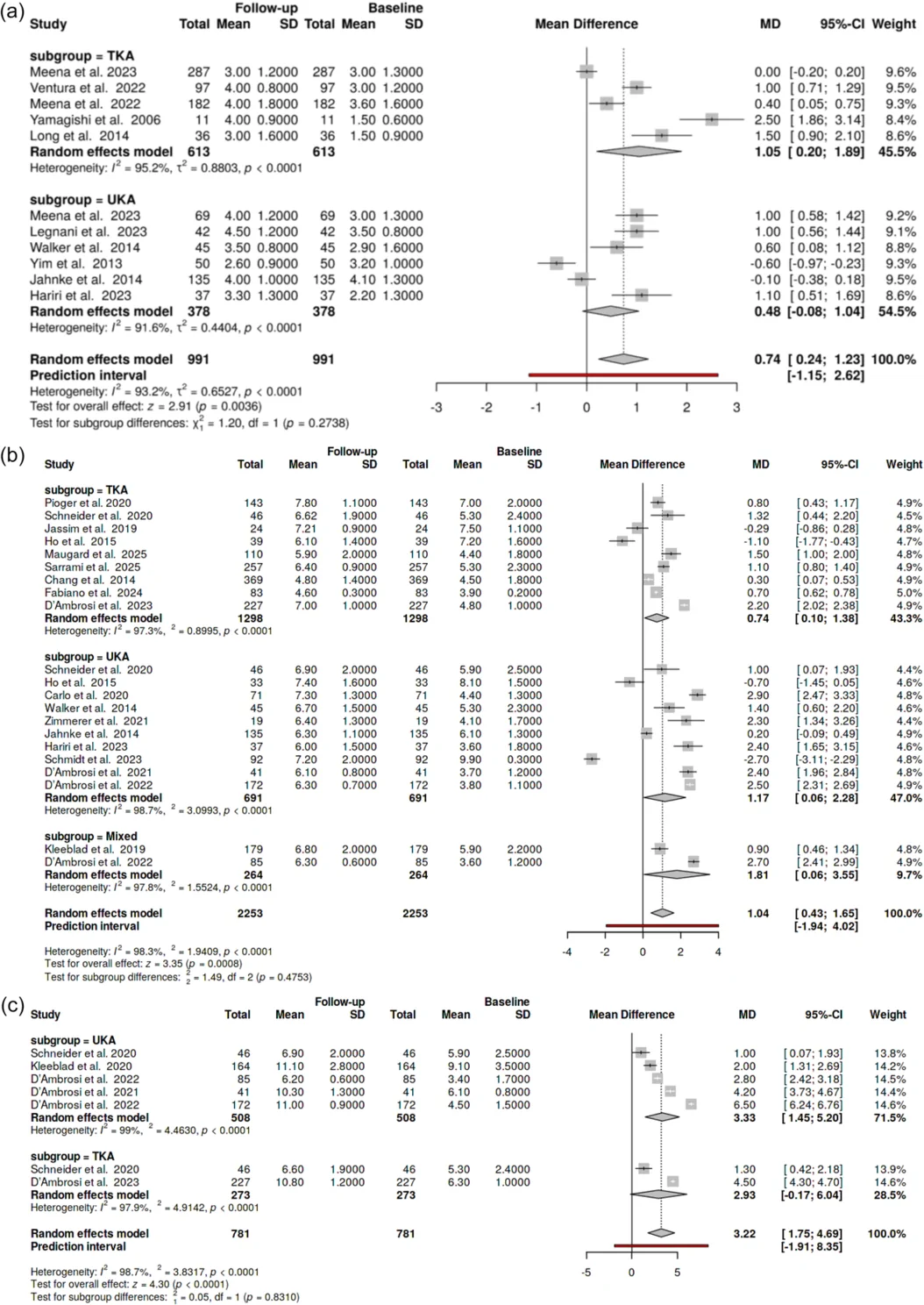
Forrest plot for pre‐to‐post on changes in PA: (a) Tegner scale; (b) UCLA scale; (c) HAAS. CI, confidence interval; HAAS, high‐activity arthroplasty score; PA, physical activity; TKA, total knee arthroplasty; UCLA, University of California, Los Angeles Activity Score; UKA, unicompartmental knee arthroplasty.

The Tegner score improved after knee arthroplasty, with a pooled MD of 0.74 points (95% CI: 0.25–1.23; *I*² = 93%). While in the TKA subgroup there was a statistically significant improvement (MD = 1.05, 95% CI: 0.20–1.89; *I*² = 95%), the improvement in the UKA subgroup was not statistically significant (Figure 5a). The funnel plot was asymmetric, suggesting risk of publication bias (Supplement S13).

The UCLA score improved significantly after knee arthroplasty, with a pooled MD of 1.04 (95% CI: 0.43–1.65; *I*² = 98%; Figure 5b). Sensitivity analysis excluding studies with non‐active participants at baseline showed that the overall and subgroups pooled effect were no longer significant (Supplement S14). Another sensitivity analysis excluding studies where the central tendency and variance were estimated retained a significant overall improvement, but not in the TKA and UKA subgroups (Supplement S15). The funnel plot showed asymmetry, suggesting risk of publication bias (Supplement S16).

The HAAS improved significantly after knee arthroplasty, with a pooled MD of 3.22 (95% CI: 1.75–4.69; *I*² = 99%). The improvement was significant in the UKA subgroup, but not in the TKA subgroup (Figure 5c). Sensitivity analysis excluding studies with non‐active participants at baseline retained a significant overall improvement, but not in the TKA and UKA subgroups (Supplement S17). Another sensitivity analysis excluding studies where the central tendency and variance were estimated showed a significant overall improvement and in both the TKA and UKA subgroups (Supplement S18).

Other PA measures—such as lower extremity activity scale (LEAS) and modified Grimby score—were reported in only a few studies and are summarised in Supplement S19.

Eleven studies [8, 21, 22, 25, 28, 29, 43, 48, 54, 57, 62] reported patient satisfaction with their post‐operative ability to engage in sports, with satisfaction rates ranging from 61% to 100% across studies. Between the TKA and UKA subgroups, the proportion of satisfaction was higher in the UKA subgroup (81% vs. 77%).

### Level of PA or sports before and after knee arthroplasty

**Figure 6:**
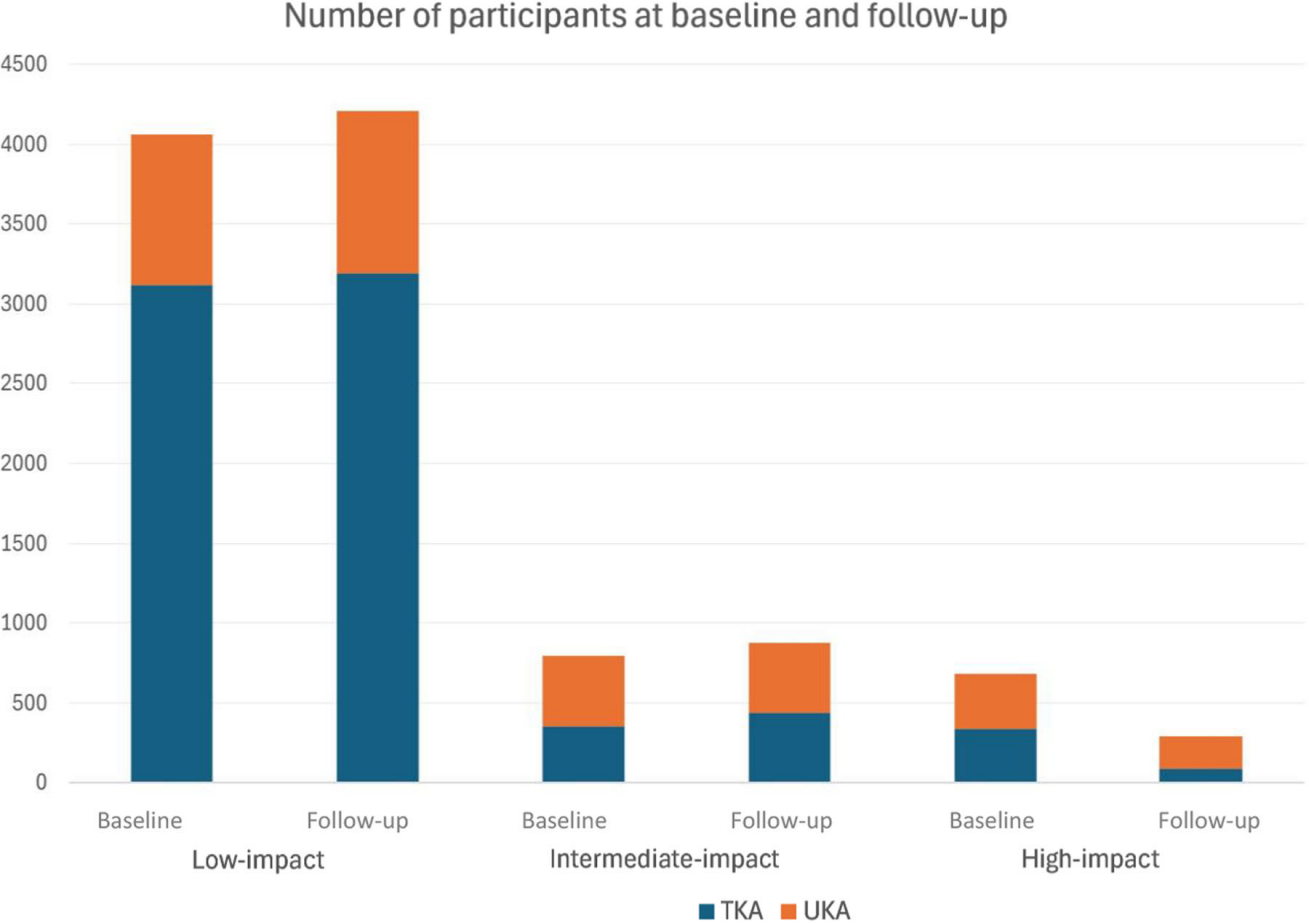
Participant in PA or sports at each impact‐level at baseline and follow‐up. PA, physical activity; TKA, total knee arthroplasty; UKA, unicompartmental knee arthroplasty.

**Figure 7:**
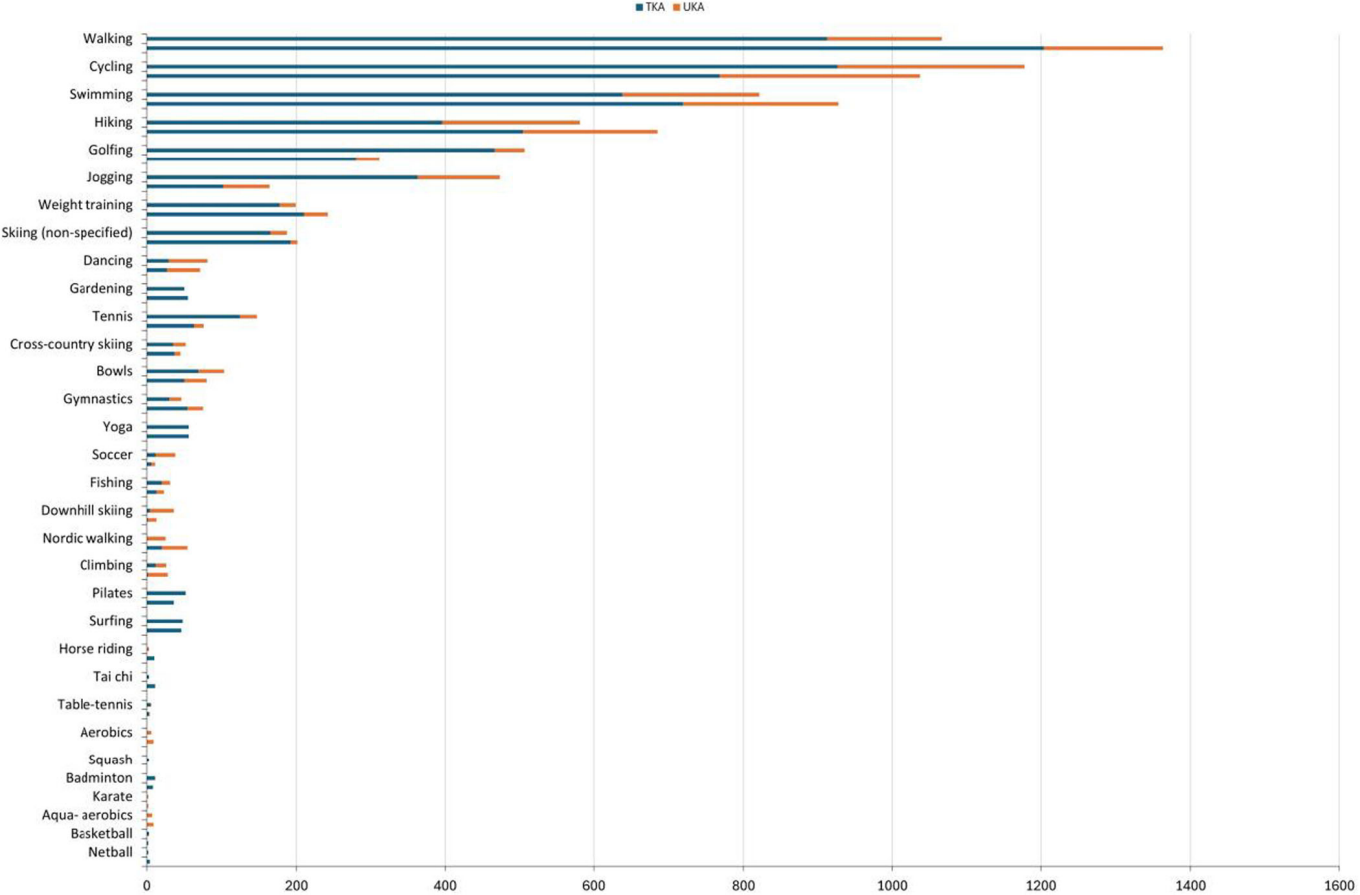
Participation in each different type of PA at baseline and follow‐up. First row of each activity indicates the participation at baseline and second row indicates the participation at follow‐up.PA, physical activity.

After knee arthroplasty, participation increased by 3.6% in low‐impact activities and by 10.3% in moderate‐impact activities, while participation in high‐impact activities decreased by 57.2% (Figure 6). The TKA subgroup showed a notable relative increase in moderate‐impact activities (+23.6%), while the UKA group had a slight decrease (−0.5%). Conversely, the TKA group experienced a larger relative decrease in high‐impact activities (−74.4%) than the UKA group (−40.3%). Low‐impact physical activities (walking, cycling and swimming) were the most common both before and after knee arthroplasty. Among the 20 most popular activities at baseline (Figure 7), Nordic walking experienced the largest relative increase (+158.8%), and soccer had the greatest relative decrease (−83.3%).

Nine studies [14, 18, 22, 23, 33, 46, 47, 57, 62] mentioned the patient′s reasons for reducing their level of sports activity. The most frequently cited reason was ‘as a precaution’ (41.6%), followed by ‘pain in other regions’ (20.5%) and ‘pain in the replaced knee’ (19.3%).

## DISCUSSION

The main finding of this systematic review is that 85% of patients successfully returned to PA following TKA and UKA. They returned typically within a relatively short timeframe (median of 4.1 months), but mostly within low‐levels of PA. No significant differences were found in the proportion of return to PA or sports between UKA and TKA. However, the comparative studies showed that those with UKA have a higher likelihood of returning to PA or sports and the return to high‐impact activities was also more common after UKA. Most patients were satisfied with their ability to participate in sports.

### Rate and time to return to PA or sports following UKA and TKA

The overall pooled proportion of return to PA or sports was high at 85%, with no significant difference between TKA (82%) and UKA (88%). This contrasts with our original hypothesis and to the findings of Papalia et al. [41], who estimated a higher return rate after UKA compared to TKA, but only included elderly patients. However, when evaluating only the comparative studies, it was found that those with UKA display a higher likelihood of returning to PA or sports. Sports participation levels also increased at follow‐up as measured by the Tegner, UCLA and HAAS scales, but it is important to note that these scales are not specifically designed to assess RTS. It is important to acknowledge that many studies included patients who were not physically active at baseline, which may affect the interpretation of these results. Previous reviews [41, 56, 59] also generally used the term ‘return to sports’ broadly, often including low‐impact physical activities that should not strictly be classified as sports, potentially misleading readers. To clarify this, the present review consistently uses the term ‘return to PA or sports,’ encompassing both low‐impact physical activities (e.g., walking, fishing, Pilates) and activities traditionally defined as sports.

The median time to return to PA or sports was 4.1 months, but with most patients resuming low‐impact activities. The median times were similar between TKA (4.1 months) and UKA (3.8 months), but only a few studies reported the time to return to PA or sports, which limits the external validity of this finds. Papalia et al. [41] reported a longer average time to return‐to‐sports of 6.2 months for UKA patients, likely influenced by older age in their cohort.

### Patient satisfaction with sports ability and reasons for sports reduction

Patient satisfaction with post‐operative sports ability was generally high, ranging from 61% to 100% across studies, with UKA patients reporting slightly higher satisfaction than TKA patients. However, these subjective perceptions and post‐operative expectations should be interpreted cautiously, as they do not provide an objective measure of actual sports capacity.

When considering overall PA levels by impact, there was an increase in participation in low‐ to intermediate‐impact activities and a decrease in high‐impact activities after knee arthroplasty. Most activities that gained participants at follow‐up were low‐impact, such as walking, swimming, and bicycling, with some exceptions like golf. Conversely, high‐impact activities including jogging and soccer showed reduced participation post‐operatively. These findings align with previous systematic reviews [41, 59] which similarly reported higher return rates to low‐impact activities and decreased engagement in high‐impact sports. Participation in high‐impact activities declined less markedly in the UKA subgroup compared to the TKA subgroup, possibly due to younger age [19, 39] and the more native knee kinematics preserved in UKA through preservation of native tissues [38]. Reasons for reduced sports participation were predominantly precautionary, followed by pain in other regions and in the replaced knee. These findings are consistent with previous reviews [56, 59], where the main reasons included ‘discouragement from surgeons’ and ‘as a way to preserve the implant, factors likely reflecting patient fear of prosthesis failure. This evidence suggests that patient beliefs regarding safe sports activity after knee arthroplasty may play a major role in their RTS, which contrasts with recent evidence highlighting the safety of returning to intermediate‐ and high‐impact physical activities in healthier and younger patients [5].

### Recommendations for clinical practice

Most patients resumed PA or sports within a few months after knee arthroplasty, providing valuable prognostic information for those aiming to stay active. While overall participation in PA or sports tends to increase postoperatively, patients are more likely to engage in low‐impact activities (e.g., swimming and cycling) rather than high‐impact ones (e.g., jogging and soccer). This difference may not solely reflect physical limitations but could also be influenced by psychological factors such as fear of prosthesis failure [22, 23, 33, 57]. Several individual factors can also influence the likelihood of returning to PA or sports after knee arthroplasty. Male sex [19, 28] and younger age [19, 39] are associated with a higher likelihood of return to pre‐operative levels of sports participation are a key determinant for resuming higher‐intensity physical activities or high‐impact sports after knee arthroplasty [28, 45]. Considering the potential advantage of UKA in better preserving the ability to participate in high‐impact activities, UKA may be the preferred option for younger patients with isolated compartment knee OA who wish to return to high‐impact sports.

### Key gaps in the literature and directions for future research

There is a notable lack of detailed description of sports activities in the current literature—as most data rely on self‐reported PA questionnaires—which hampers the ability to objectively categorise patients′ levels of sports participation. While questionnaires provide valuable insights into self‐reported functional ability, they are often limited to ceiling effects. Therefore, there is a need for more objective assessment tools that are focused specifically on sports participation to improve the accuracy and relevance of future research in this field.

Another major limitation in the current literature is the insufficient reporting of PA restrictions based on physician recommendations. Given the critical role that these factors have in returning to sports, future studies should aim to provide more detailed descriptions of guidance or restrictions advised by physicians.

Future studies should improve the reporting of return to PA or sports by adopting more precise and standardised definitions. It is essential to clearly distinguish between return to low‐impact and high‐impact PA, as these represent different levels of engagement and intensity. Studies should report the number of patients who returned to sports specifically from those who were active in sports before surgery, rather than presenting averages that combine both active and sedentary individuals pre‐ and post‐operatively. Lastly, the timing of the pre‐operative assessment of PA or sports participation is often vaguely described as ‘before surgery,’ which lacks specificity and can be misleading regarding the actual reference point for baseline activity levels. Since knee OA typically causes a gradual decline in sports activity, some studies have used the time before the onset of knee symptoms as the baseline instead of the time immediately preceding surgery. To enhance comparability across studies and provide more accurate prognostic information, there is a clear need for standardised reporting on the timing of baseline PA or sports assessments.

### Limitations of the present systematic review

The present systematic review has several limitations. Although the present review included 45 studies, the number of comparative studies between UKA and TKA was limited, restricting direct comparisons of many outcomes between these subgroups. Despite including a meta‐analysis (which most previous systematic reviews did not), there was high risk of bias and the certainty of evidence was very low, and therefore our findings should be read with caution. Many studies included non‐active, older and obese patients, which may not accurately represent the physically active population and could introduce selection bias and limit external validity. Third, the classification of patients as active or nonactive was often based on poorly described PA status, leading to potential misclassification of individuals with only occasional sports participation as ‘active’, which introduces a risk of selection bias. Moreover, there were often lacking the rates of patients that returned to PA or sports from those that were physically active or not active at baseline. Most studies failed to provide information about comorbidities, which could influence return to PA or sports. There was also inconsistent and often vague timing of pre‐operative PA assessments, complicating the comparison of pre‐ and post‐operative activity levels. Furthermore, many studies did not provide comprehensive details on rehabilitation protocols or physician recommendations, which are important factors influencing RTS. Finally, the reliance on self‐reported PA questionnaires limited the objectivity of sports participation measures, and publication bias may have influenced the overall findings.

## CONCLUSION

The rate of return to PA or sports following knee arthroplasty was high (93%) and reporting high levels of satisfaction. Most patients resuming PA within a relatively short timeframe, but returned mostly to low‐impact activities. Participation in high‐impact activities decreased post‐operatively, suggesting some level of restriction or caution regarding engagement in more demanding sports. Importantly, the primary reason cited for reduced sports involvement was concern over prosthesis failure, highlighting that psychological factors and patient perceptions may play a significant role in limiting high‐impact activity, beyond actual physical capability. Therefore, patients can expect return to low‐impact PA within a relatively short timeframe in most cases, but RTS may be compromised.

## AUTHOR CONTRIBUTIONS

All authors were involved both in the idealisation of the systematic review and preparation of the manuscript. Rodrigo Braz and Renato Andrade were involved in the databases searches and data extraction. Rodrigo Braz, Renato Andrade and Cristina Valente performed all data analysis and interpretation of results. Rodrigo Braz and Renato Andrade judged the risk of bias of the studies included in the systematic review. Renato Andrade and Eluana Gomes evaluated the certainty of evidence. Rodrigo Braz, Renato Andrade and Eluana Gomes prepared the first draft of the paper. All authors contributed to revise subsequent drafts and approve the final manuscript prior to submission to the peer‐reviewed journal.

## CONFLICT OF INTEREST STATEMENT

The authors declare no conflicts of interest.

## ETHICS STATEMENT

The authors have nothing to report.

## Supporting information

Supplementary Information

## Data Availability

All data are presented in the manuscript or in supplementary files.
