# Supplementary material for: High rate of return to low‐impact physical activity or sports after total and unicompartmental knee arthroplasty: A systematic review with meta‐analysis
Source: Knee Surg Sports Traumatol Arthrosc. 2026 Jan 13;34(9):3415–28. doi: 10.1002/ksa.70267 (PMC13502459; doi:10.1002/ksa.70267)
Supplement: Supplementary file 1 [file KSA-34-3415-s001.pdf]

LIST OF SUPPLEMENTS

Supplement 1 - Deviations from protocol.

Supplement 2 – Search strategy for each database.

Supplement 3 - Data collection and data management.

Supplement 4 - Risk of Bias Assessment tool for Non-randomized Studies (RoBANS).

Supplement 5 - Grading of Recommendations Assessment, Development and Evaluation (GRADE) evaluation.

Supplement 6 - Study and population characteristics.

Supplement 7 - Surgical procedures characteristics.

Supplement 8 - Additional details on the arthroplasty procedure.

Supplement 9 - Return to PA or sports after knee arthroplasty.

Supplement 10 - Sensitivity analysis for return to PA or sports proportion, excluding studies with ambiguous assessment of return to PA or sports.

Supplement 11 - Funnel plot to assess the risk of publication bias for return to PA or sports proportion.

Supplement 12 - Sensitivity analysis between-studies odds ratio (OR) of return to PA or sports proportion, excluding studies with ambiguous assessment of return to PA or sports.

Supplement 13 - Funnel plot to assess the risk of publication bias for pre-to-post Tegner score.

Supplement 14 - Sensitivity analysis for pre-to-post UCLA score, excluding studies with pre-operative non-active participants.

Supplement 15 - Sensitivity analysis for pre-to-post UCLA score, excluding studies with estimated central tendency and variance data.

Supplement 16 - Funnel plot to assess risk of publication bias for UCLA scores.

Supplement 17 - Sensitivity analysis for pre-to-post HAAS, excluding studies with pre-operative non-active participants.

Supplement 18 - Sensitivity analysis for pre-to-post HAAS, excluding studies with estimated central tendency and variance data.

Supplement 19 - PA scores at baseline and follow-up.

List of references

Supplement 1 - Deviations from protocol.

Although initially intended to categorize patients using the Participant Classification Framework (PCF) [30], several limitations arose while trying to translate the PCF classification to our cohort. Firstly, the PCF is suitable for younger individuals (18-65 years), making it unreliable for the older participants included in this systematic review. Secondly, PCF's detailed sports characterization (e.g., weekly sessions, types of sports, competition status) was often not available, as the included studies did not assess sports participation to this extent. Consequently, patients were instead classified as either physically active or non-active as reported above. For the same reasons, and in addition due to the poor reporting and heterogeneity in the population across studies, it was decided to include all studies that report the rate or time to return to physical activity or sports after knee arthroplasty, regardless of the level of activity at pre-surgery.

Supplement 2 – Search strategy for each database.

Databases	Results	Search strategy	Date
Pubmed	1,456	(knee OR tibiofemoral OR femorotibial) AND (total OR bicompartmental OR bicondylar OR unicondylar OR unicompartmental OR partial) AND (arthroplasty OR replacement OR prosthesis) AND (sport* OR play OR athlet* OR “physical activity” OR “physical exercise” OR competition OR exercise) AND (returning OR resuming OR return OR resume OR “coming back” OR restarting OR restoration OR retraining OR reestablishment)	28/11/2025
Web of science (core collection)	855	(knee OR tibiofemoral OR femorotibial) AND (total OR bicompartmental OR bicondylar OR unicondylar OR unicompartmental OR partial) AND (arthroplasty OR replacement OR prosthesis) AND (sport* OR play OR athlet* OR “physical activity” OR “physical exercise” OR competition OR exercise) AND (returning OR resuming OR return OR resume OR “coming back” OR restarting OR restoration OR retraining OR reestablishment)	28/11/2025
EMBASE	944	(knee OR tibiofemoral OR femorotibial) AND (total OR bicompartmental OR bicondylar OR unicondylar OR unicompartmental OR partial) AND (arthroplasty OR replacement OR prosthesis) AND (sport* OR play OR athlet* OR 'physical activity' OR 'physical exercise' OR competition OR exercise) AND (returning OR resuming OR return OR resume OR 'coming back' OR restarting OR restoration OR retraining OR reestablishment)	28/11/2025
**Total**	**3,255**		

Supplement 3 - Data collection and data management.

Data collection

(i) *Study characteristics* - author, study reference, year of publication, study design, sample size and post-operative follow-up time (months).

(ii) *Patients characteristics* - age, sex (male or female), body mass index (BMI), number of knees and knee laterality (right, left).

(iii) *Intervention-related characteristics* - type of arthroplasty (UKA or TKA), if UKA (medial or lateral side), navigation method (traditional or robotic), prosthesis brand or type and other details about the arthroplasty procedure (e.g., if cruciate ligaments are retained or not).

(iv) *Outcomes of interest* - rate of return to PA or sports (as number and percentage), time to return to PA or sports (months), time of pre-operative sports assessment (e.g., before surgery, before the onset of the first restricting symptoms) and the definition of return to PA or sports. The PA scores (e.g., Tegner scale, University of California, Los Angeles Activity Score [UCLA] or High-Activity Arthroplasty Score [HAAS]), number of physically active patients, frequency and length of sports participation and number and types of sports practiced were also compiled at pre- and post- knee arthroplasty. The patient satisfaction with their sports ability and reasons for sports reduction were also collected.

Data management

Continuous data was collected as mean and standard deviation (SD), and categorical data as number and percentage (%). We prioritized the extraction of mean and SD, but in instances where mean and SD were not provided, it was sought to calculate these metrics (when feasible) using the methods recommended by the Cochrane Handbook [5]; otherwise, any other metric of central tendency was collected. When data was presented as median and range, the mean and SD were estimated according to Hozo et al. [18]. When data was presented in figures or graphs, the WebPlotDigitizer online tool was employed to extract the relevant outcome data.

Whenever possible, outcomes were extracted at both the baseline and follow-up stages. In cases where there was more than one follow-up endpoint, the data at the last

follow-up was used. When only the minimum value was presented (e.g., follow-up ≥12), it was considered the minimum values for the estimation of weighted mean. If only the range was given, the midpoint of the range was calculated and considered for the weighted mean.

The estimation of included patient's sex distribution did not consider studies that reported sex count based on knees or accounted for patients that did not meet eligibility criteria for inclusion in the present systematic review.

For the classification of patients as physically active, only those who competed or participated at any number of sports were considered. However, if the sports participation was reported as rare or occasional, the patient was classified as non-active. The PA was classified as high-, intermediate- and low- impact on the knee joint; when the level of impact was not provided by the study, it was classified based on Vail et al. [43]:

**Low-impact**	**Intermediate-impact**	**High-impact**
Walking	Hiking	Jogging
Cycling	Downhill skiing	Soccer
Swimming	Horse riding	
Golfing		
Cross-country skiing		
Dancing		
Bowls		
Aqua aerobics		
Table-tennis		

The assessment of patient's satisfaction with ability to practice sports after knee arthroplasty was categorized dichotomously as satisfied and not satisfied. Satisfied included those reporting as "satisfied" and "very satisfied", or when sports performance was reported to have improved or stayed the same, or when sports performance classified as "fair", "good" and "excellent".

Supplement 4 - Risk of Bias Assessment tool for Non-randomized Studies (RoBANS).

Domain	Description
**Selection of participants**	**Selection bias** caused by inadequate selection of participants.<br><br>Selection of participants must include individuals with primary or post-traumatic knee OA. The participants selected must have been treated with UKA or TKA.<br><br>Is considered as high risk including in the same sample:<br>- Individuals with traumatic and secondary non-traumatic knee OA.<br>- Individuals selected based on post- surgery PA levels.<br>- Individuals that were sedentary before surgery.
**Confounding variables**	**Selection bias** caused by inadequate confirmation and consideration of confounding variable.<br><br>Sample of participants should be homogenous in terms of confounding variables:<br><br>- *Patient-related*: age, sex, BMI, presence of comorbidities, level of preoperative knee OA and extent of preoperative participation in physical exercise.<br>- *Intervention-related*: prosthesis type, use of navigation system, cemented status, modality of prosthesis bearing, cruciate ligament preservation and rehabilitation protocol.<br>- *Outcome:* time of pre-operative physical activity assessment and duration of follow-up.<br><br>When including comparison groups (and considered for the systematic review), the groups must be homogenous in regards to confounding variables.
**Exposure measurement**	**Performance bias** caused by inadequate measurement of exposure/ outcome.<br><br>Clinical and patient-reported outcomes must be collected using validated methods. The time to follow-up should be at least 24 months.<br><br>- Physical activity- related outcomes must be clearly described. If the methods of measurement (e.g. return to sports definition and activity level classification) are poorly described and do not allow replication, then the study is judged with high risk.<br><br>- Patient-reported outcomes must be collected prospectively to prevent recall bias.
**Blinding outcome assessment**	**Detection bias** caused by inadequate blinding of outcome assessment.<br><br>Assessor and/or data analysist not blinded intervention.
**Incomplete outcome data**	**Attrition bias** caused by inadequate handing of incomplete data outcome.<br><br>Missing data in >5% of outcome variable.
**Selective outcome reporting**	**Reporting bias** caused by selective outcome reporting.<br><br>Based on reporting of the collected/assessed outcomes and statistical analysis.<br><br>Is consider as high risk:<br>- Studies where the methods provided no information on how the analyses would be performed.<br>- Studies with significant attrition bias and the reasons for missing data were unreported.

Supplement 5 - Grading of Recommendations Assessment, Development and Evaluation (GRADE) evaluation.

**Outcome**	**Risk of bias**	**Inconsistency**	**Imprecision**	**Indirectness**	**Publication bias**	**Overall certainty**	**Figure**
Proportion of patients who returned to PA or sports	Serious risk of bias *	Serious inconsistency †	Low risk of imprecision	Serious indirectness #	Serious risk of publication bias §	Very low ⊕◯◯◯	Fig. 3
Between-group OR (TKA vs UKA) to return to PA or sports	Serious risk of bias *	Low risk of inconsistency	Very serious imprecision ‡	Serious indirectness #	*Not applicable*	Very low ⊕◯◯◯	Fig. 4
Pre-to-post MD for Tegner scale	Serious risk of bias *	Serious inconsistency †	Low risk of imprecision	Low risk of indirectness	Serious risk of publication bias §	Very low ⊕◯◯◯	Fig. 5A
Pre-to-post MD for UCLA scale	Serious risk of bias *	Serious inconsistency †	Low risk of imprecision	Low risk of indirectness	Serious risk of publication bias §	Very low ⊕◯◯◯	Fig. 5B
Pre-to-post MD for HAAS	Serious risk of bias *	Serious inconsistency †	Low risk of imprecision	Low risk of indirectness	*Not applicable*	Very low ⊕◯◯◯	Fig. 5C

* Serious risk of bias due to one or more studies with high risk of bias

† Serious inconsistency due to substantial statistical heterogeneity or asymmetric point estimates

‡ Very serious imprecision due to insufficient sample size (n<800) and wide confidence intervals

Serious indirectness due to heterogeneity in determining the outcome

§ Serious risk of publication bias as evaluated with visual inspection of funnel plots

Supplement 6 - Study and population characteristics.

Author name, year	Sample (knees)	Age (years)	Sex, F:M	BMI (kg/m²)	Follow-up, mean ± SD (months) α
Pioger et al. (2020) [35]	143 (143)	65.7 ± 7.2	21:122	N.R.	55.45 ± 36.9
Schneider et al. (2020) [39] §	115 (115)	56 ± 9.1	100:15	28 ± 5.6	12-24
Plassard et al. (2020) [36]	443 (443)	69 (41-90)	281:162	29.3 (19–46)	43 (23–49)
Meena et al. (2023) [31]	356 (356)	**TKA:** 75.7 ± 8.1	141:146	26.5 ± 3.8	24
		**UKA:** 74.2 ± 8.8	27:42	26.8 ± 3.2	
Naylor et al. (2019) [32]	718 (733)	**Physically active:** 67.8 ± 8.2	116:602	**Physically active:** 31.7 ± 6.3	36
		**Non-physically active:** 69.0 ± 8.9		**Not physically active:** 33.0 ± 6.9	
Hopper et al. (2008) [17]	110 (110)	**TKA:** 62.1 ± 8.9 (35–75)	44:32	N.R.	21.6 ± 5.3 (13–42)
		**UKA:** 61.3 ± 9.0 (43–75)	14:20	N.R.	22.3 ± 7.8 (12–44)
Walton et al. (2006) [46]	270 (325)	**TKA:** 71.5 ± 9.9 (9-95)	61:59	N.R.	≥12
		**UKA:** 71.5 ± 9.8 (36-92)	74:76	N.R.	≥12
Tramer et al. (2022) [42]	54 (54)	**RTS:** 66.6 ± 7.3	8:36	32.3 ± 6.3	12.6 ± 6.3
		**No RTS:** 66.2 ± 5.8	3:7	31.9 ± 5.5	
Jassim et al. (2019) [21]	24 (24)	60 ± 2.5 (54-64)	14:10	N.R.	36 ± 10.8 (24-54)
Cozzarelli et al. (2024) [4]	296 (296)	**RTS:** 60.7 ± 9.5	113:102	27.9 ± 4.1	≥12
		**No RTS:** 62.6 ± 10.7	44:37	28.7 ± 4.5	
Ho et al. (2015) [16]	72 (76)	**TKA:** 59.3 (56.6–63.6)*	28:12 ϕ	32.5 (28.8–38.4)*	50.4 ± 14.4 (26.4–74.4)
		**UKA:** 60.0 (53.3–63.6)*	21:12 ϕ	30.3 (27.6–33.7)*	45.6 ± 13.2 (24–68.4)
Carlo et al. (2022) [2]	137 (137)	54.3 (47–60)	48:89	27.8 ± 2.5	74.4 (62.2-90)
Lo Presti et al. (2019) [26]	53 (53)	59.7 (46–66)	38:15	≤30	48 ± 6 (24–72)
Legnani et al. (2023) [25]	42 (42)	67.8 ± 2.5	18:24	25.1 ± 1.5	38.4 ± 18
Walker et al. (2015) [45]	45 (45)	60.1 ± 10.5 (36–81)	26:19	**Female:** 26 (21–42) Ɨ<br>**Young:** 26 (21–42) Ɨ<br>**Old:** 28 (22–36) Ɨ	35.4 ± 8.1 (24–51)

Kleeblad et al. (2020) [23]	164 (179)	62.3 ± 8.8 (33.2–87.3)	74:90	27.6 ± 4.4 (18.3–43.4)	20.2 (12.0–31.0)
Ventura et al. (2022) [44]	97 (97)	70.1 (64–83)	54:43	23.5 ±1.6	49.1 ± 13 .2
Pietschmann et al. (2013) [34]	131 (131)	65.3 (44–90)	74:57	27.6 ± 4.7 (20–56)	50.4 (12–120)
Zimmerer et al. (2021) [49]	19 (19)	56.7 ± 10.5 (36.0–76.0)	14:5	27.2 ± 5.7 (17.3–38.0)	55.2 ± 26.4 (24.0–116.4)
Hepperger et al. (2018) [15]	200 (235)	72.2 ± 7.7	120:80	27.2 ± 5.0	24
Kim et al. (2019) [22]	42 (42)	63.6 ± 5.5 (54–79)	35:7	25.3 ± 2.4 (20.7–30.8)	≥24
Yim et al. (2013) [48]	50 (50)	60.3 (47-65)	48:2	N.R.	44.4 (36-48)
Yamagishi et al. (2006) [47]	11 (11)	68 (47-76)	4:7	N.R.	26.4 (11 to 60)
Huch et al. (2005) [19]	389 (389)	66.3 ± 6.4	283:106	<25: 55 patients<br>25-29: 175 patients<br>≥30: 159 patients	60
Jahnke et al. (2015) [20]	135 (147)	63.5 (36–86) #	75:84 #	N.R.	24 ± 17.6
Deng et al. (2023) [11]	75 (75)	**BKA**: 60.0 ± 8.0	**BKA**: 11:14	**BKA**: 26.1 ± 3.0	**BKA**: 57.6 (27.6-62.4) *
		**TKA**: 62.2 ± 6.1	**TKA**: 22:28	**TKA**: 26.5 ± 2.5	**TKA**: 55.2 (27.6-67.2) *
Daffara et al. (2024) [10]	136 (148)	69.0 ± 8.5	83:53	N.R.	72.0 ± 16.8
D'Ambrosi et al. (2022) [9]	85 (85)	**Medial UKA:** 60.3 ± 4.9	**Medial UKA:** 58:15	**Medial UKA:** 26.6 ± 3.3	**Medial UKA:** 41.6 ± 15.1
		**Lateral UKA:** 60.7 ± 6.0	**Lateral UKA:** 7:5	**Lateral UKA:** 26.4 ± 1.7	**Lateral UKA:** 42.0 ± 16.6
Hariri et al. (2023) [14]	37 (37)	52.8 ± 3.1	22:15	30.0 ± 6.8	37.2 ± 18
Schmidt et al. (2023) [38]	92 (92)	64.9 ± 6.4	32:60	27 ± 3.5	87.6 ± 20.4
Antonelli et al. (2023) [1]	2477 (2477)	65.2 ± 10.8 #	2341:1698 #	29.4 ± 6.2 #	12.4 (1.5-75) #
Maugard et al. (2025) [29]	110 (110)	66.9 ± 8.9	70:40	29.3 ± 5.9	40.8 ± 6.3
Sarrami et al. (2025) [37]	257 (336)	68.0 ± 7.2	129:128	29.6 ± 5.2	≥12
Taylor et al. (2025) [41]	382 (382)	66.0 ± 10.1 #	680:604 #	29.3 ± 6.2 #	42.3 ± 31.2 #
Lombardi et al. (2009) [27]	206 (230)	**TKA**: 62 ± 10.0	**TKA**: 65:38	**TKA**: 31 ± 5.1	**TKA**: 32 ± 10.4
		**UKA**: 61 ± 10.3	**UKA**: 65:38	**UKA**: 31 ± 5.1	**UKA**: 30 ± 12.1
Driesman et al. (2024) [12]	332 (332)	69.0 ± 9.4 #	553:476 #	28.0 ± 5.6 #	49 ± 36 #
Lawrence et al. (2024) [24]	784 (784)	62.8 ± 2.2	338:446	29.8 ± 0.8	58.8 ± 34.8
Peters et al. (2024) [33]	2063 (2063)	65.8 ± 7.2	1090:973	27.8 ± 3.4	6.0
Chang et al. (2014) [3]	369 (620)	68.8 (50-83)	339:30	27.4 (19.3-39.1)	≥12
Fabiano et al. (2024) [13]	83 (83)	68.1 (50–85)	55:28	N.R.	12

Streck et al. (2023) [40]	1636 (1907)	**TKA:** 64 ± 7<br>**UKA:** 62 ± 7	1083:824	**TKA:** 31.3 ± 6.5<br>**UKA:** 29.3 ± 6.5	≥24
D'Ambrosi et al. (2021) [8]	41 (41)	66.4 ± 6.5	33:8	28.0 ± 3.6	67.0 ± 18.2
D'Ambrosi et al. (2023) [7]	227 (227)	59.9 ± 2.1	150:77	N.R.	64.0 ± 4.3
D'Ambrosi et al. (2022) [6]	172 (172)	68.8 ± 2.7	108:64	27.9 ± 2.0	≥24
Long et al. (2014) [28]	36 (45)	51 #	N.R.	N.R.	301 (240–420)

Legend: M Male; **F** Female; **L** Left; **R** Right; **N.R**. Not reported; **RTS** Return to sport; **UKA** unicompartmental knee arthroplasty; **TKA** total knee arthroplasty; **BKA** bicompartmental knee arthroplasty

* Median [interquartile]. Ɨ Median (range). # According to the total sample and not the sample included in the systematic review. ǂ According to the number of patients and not knees. **ϕ** According to the number of knees and not patients. **§** Takes into consideration 23 patellofemoral patients. **α** The follow-up time begins at the time of surgery.

Supplement 7 - Surgical procedures characteristics.

First author, year	Type of arthroplasty, patients	Navigation system?	Prosthesis brand/type
Pioger et al. (2020) **[35]**	TKA (n=143)	No	N.R.
Schneider et al. 2020) **[39]** *	TKA (n=46)<br>UKA (n=46)	No	N.R.
Plassard et al. (2020) **[36]**	TKA (n=443)	No	N.R.
Meena et al. (2023) **[31]**	TKA (n=287)<br>UKA (n=69)	No	**TKA:** NexGen CR, Zimmer Inc.<br><br>**UKA:** Oxford mobile-bearing prosthesis (Biomet, Inc., Warsaw, IN)
Naylor et al. (2019) **[32]**	TKA (n=718)	No	N.R.
Hopper et al. (2008) **[17]**	TKA (n=76)<br><br>UKA (n=34)	No	**TKA:** total condylar design, the majority of which were Press-Fit Condylar (DePuy, Warsaw, Indiana)<br>**UKA:** Oxford designed implant
Walton et al. (2006) **[46]**	TKA (n=120)<br>UKA (n=150)	No	N.R.
Tramer et al. (2022) **[42]**	TKA (n=54)	No	N.R.
Jassim et al. (2019) **[21]**	TKA (n=24)	No	N.R.
Cozzarelli et al. (2024) **[4]**	Medial UKA (n=278)<br>Non-specific UKA (n=18)	134 UKA: with robotic assistance.<br>162 UKA: without robotic assistance.	N.R.
Ho et al. (2015) **[16]**	TKA (n=39)<br>UKA (n=33)	No	N.R.
Carlo et al. (2022) **[2]**	Medial UKA (n=137)	No	Oxford unicompartmental knee prostheses (Biomet UK Ltd., Bridgend, United Kingdom)
Lo Presti et al. (2019) **[26]**	Medial UKA (n=53)	No	Uni Preservation prosthesis (DePuy International Ltd., Leeds, United Kingdom)
Legnani et al. (2023) **[25]**	UKA (n=42)	No	Oxford unicompartmental knee prosthesis (Zimmer-Biomet, Warsaw, IN, United States of America)
Walker et al. (2015) **[45]**	Lateral UKA (n=45)	No	Oxford domed lateral component (Biomet UK Ltd, Swindon, United Kingdom).
Kleeblad et al. (2020) **[23]**	UKA (n=164)	With robotic assistance.	RESTORIS MCK Onlay tibial implant (Stryker Corp., Mahwah, NJ).

Ventura et al. (2022) **[44]**	TKA (n=97)	No	Posterior stabilized Vanguard Complete Knee System (Zimmer-Biomet Orthopedics, Warsaw, IN, United States of America)
Pietschmann et al. (2013) **[34]**	Medial UKA (n=131)	No	Oxford Phase III unicompartmental knee prosthesis (Biomet, Bridgend, United Kingdom).
Zimmerer et al. (2021) **[49]**	Lateral UKA (n=19)	No	ZUK (Fa. Zimmer Inc., Warsaw, IN, USA) and the Sigma Partial Knee (DePuy Synthes, Warsaw, IN, United States of America) were used in 10 and 9 patients, respectively.
Hepperger et al. (2018) **[15]**	TKA (n=200)	No	Total knee implant (NexGen CR, Zimmer Inc.).
Kim et al. (2019) [22]	UKA (n=42)	No	Phase III Oxford UKA guided by the Microplasty® instrumentation system (Zimmer Biomet, Warsaw, IN, United States of America).
Yim et al. (2013) **[48]**	UKA (n=50)	No	Miller-Galante prosthesis (Zimmer, Warsaw, Indiana).
Yamagishi et al. (2006) **[47]**	TKA (n=11)	No	3 patients: Wright Advantim knee prosthesis.<br>8 patients: Wright Advance system (Wright Medical, United States of America).
Huch et al. (2005) **[19]**	TKA (n=389)	No	N.R.
Jahnke et al. (2015) **[20]**	Medial UKA (n=135)	No	N.R.
Deng et al. (2023) **[11]**	BKA (n=25)<br><br>TKA (n=50)	No	**BKA**: Medial fixed-bearing unicompartmental implant (Zimmer, Warsaw, IN, USA) and an onlay patellofemoral implant (Zimmer, Warsaw, IN, USA)<br>**TKA**: Genesis II posterior-cruciate-retaining knee arthroplasty system (Smith & Nephew, Memphis, TN, USA)
Daffara et al. (2024) **[10]**	UKA (n=136)	With robotic assistance.	Fixed-bearing, cemented metal-backed implants [Restoris MCK, Mako Surgical Corp. (Stryker), Fort Lauderdale, FL, USA]
D'Ambrosi et al. (2022) **[9]**	Medial UKA (n=73)<br>Lateral UKA (n=12)	No	**Medial UKA**: Oxford Microplasty instrumentation (Zimmer Biomet, Warsaw, Indiana, USA).<br>**Lateral UKA**: Physica ZUK (Lima Corporate, Udine, Italy)
Hariri et al. (2023) **[14]**	UKA (n=37)	No	Oxford Fixed Lateral prosthesis (Zimmer Biomet UK, Bridgend)
Schmidt et al. (2023) **[38]**	UKA (n=92)	No	Zimmer® Unicompartmental High Flex System, Warsaw, Indiana, USA
Antonelli et al. (2023) **[1]**	TKA (n= 2288)<br>UKA (n= 189)	N.R.	N.R.
Maugard et al. (2025) **[29]**	TKA (n=110)	No	Primary posterior-stabilized cemented implant (Anatomic®, Amplitude)
Sarrami et al. (2025) **[37]**	TKA (n=257)	No	N.R.
Taylor et al. (2025) **[41]**	TKA (n=326)<br>UKA (n=56)	N.R.	N.R.
Lombardi et al. (2009) **[27]**	TKA (n=115)<br>UKA (n=115)	No	**TKA**: Vanguard cruciate retaining prosthesis (Biomet Inc)

			**UKA**: Oxford Phase III mobile-bearing unicompartmental knee prosthesis (Biomet Inc, Warsaw, Indiana)
Driesman et al. (2024) **[12]**	TKA (n=332)	N.R.	N.R.
Lawrence et al. (2024) **[24]**	TKA (n=784)	N.R.	N.R.
Chang et al. (2014) **[3]**	TKA (n=369)	N.R.	N.R.
Fabiano et al. (2024) **[13]**	TKA (n=83)	N.R.	N.R.
Streck et al. (2023) **[40]**	TKA (n=1746)<br>UKA (n=161)	89 UKA: robotic assisted	N.R.
D'Ambrosi et al. (2021) **[8]**	UKA (n=41)	No	TiNbN hypoallergenic medial UKA Oxford (Zimmer-Biomet, Warsaw, Indiana, USA)
D'Ambrosi et al. (2023) **[7]**	TKA (n=227)	No	Vanguard® Knee System (Zimmer Biomet, Warsaw, IN, USA)
D'Ambrosi et al. (2022) **[6]**	UKA (n=172)	No	Cobalt-Chromium UKA with the Oxford Microplasty instrumentation (n=136)<br>Anallergic Titanium Niobium Nitride medial UKA (n=36)
Long et al. (2014) **[28]**	TKA (n=36)	N.R.	N.R.

Legend: N.R. Not reported; **UKA** unicompartmental knee arthroplasty**; TKA** total knee arthroplasty; **CR** cruciate retaining; **BKA** bicompartmental knee arthroplasty.

*Takes into consideration 23 patellofemoral patients.

Supplement 8 - Additional details on the arthroplasty procedure.

First author, year	With tourniquet?	Approach	Patella resurfaced?	Cruciate retaining?	Cemented?	Mobile-bearing?
Plassard et al. (2020) [36]	No	**Varus Knees:** medial parapatellar trans-quadricipital<br>**Valgus knees:** Keblish lateral	When severe patellar OA was present	N.R.	N.R.	N.R.
Meena et al. (2023) [31]	N.R.	Medial parapatellar	NR	**TKA:** yes<br><br>**UKA:** N.R.	**TKA:** yes<br><br>**UKA:** N.R.	**TKA:** N.R.<br><br>**UKA:** yes
Hopper et al. (2008) [17]	N.R.	N.R.	N.R.	N.R.	N.R.	N.R.
Walton et al. (2006) [46]	N.R.	**TKA:** medial parapatellar approach with patellar eversion.<br>**UKA:** mini-medial arthrotomy without patellar eversion.	N.R.	N.R.	**TKA:** Cemented, uncemented, and hybrid prostheses were used according to surgeon preference<br>**UKA:** N.R.	N.R.
Carlo et al. (2022) [2]	N.R.	N.R.	N.R.	N.R.	Yes	Fixed-bearing
Lo Presti et al. (2019) [26]	N.R.	Minimally invasive quadriceps-sparing technique	N.R.	N.R.	Yes	N.R.
Legnani et al. (2023) [25]	N.R.	Minimally invasive medial parapatellar approach with a short vertical incision from the medial border of the patella to the medial edge of the anterior tibial tuberosity	N.R.	N.R.	Yes	N.R.

		without dislocation of the patella				
Walker et al. (2015) [45]	N.R.	N.R.	N.R.	N.R.	N.R.	Yes
Kleeblad et al. (2020) [23]	N.R.	N.R.	N.R.	N.R.	Yes	Fixed- bearing
Ventura et al. (2022) [44]	N.R.	Implanted using medial parapatellar arthrotomy	No	N.R.	Yes	N.R.
Pietschmann et al. (2013) [34]	N.R.	Minimally invasive antero-medial approach	N.R.	N.R.	N.R.	N.R.
Zimmerer et al. (2021) [49]	N.R.	N.R.	N.R.	N.R.	N.R.	Yes
Hepperger et al. (2018) [15]	Yes	Medial parapatellar.	N.R.	Yes	Yes	N.R.
Kim et al. (2019) [22]	N.R.	N.R.	N.R.	N.R.	Yes	Yes
Yim et al. (2013) [48]	N.R.	N.R.	N.R.	N.R.	Yes	Fixed-bearing
Yamagishi et al. (2006) [47]	Yes	Medial subvastus.	N.R.	N.R.	No	N.R.
Deng et al. (2023) [11]	Yes	Medial parapatellar retinacular	Yes (11 cases in BKA and 1 case in TKA)	Yes	N.R.	Fixed-bearing

Daffara et al. (2024) [10]	N.R.	Minimally invasive mid-vastus approach	N.R.	N.R.	Yes	Fixed-bearing
D'Ambrosi et al. (2022) [9]	N.R.	N.R.	N.R.	N.R.	N.R.	N.R.
Hariri et al. (2023) [14]	N.R.	Lateral parapatellar approach	N.R.	N.R.	**Tibial component**: always<br>**Femoral component**: depending on the bone quality	N.R.
Schmidt et al. (2023) [38]	Yes	N.R.	N.R.	N.R.	Yes	Fixed-bearing
Antonelli et al. (2023) [1]	N.R.	N.R.	N.R.	N.R.	N.R.	N.R.
Maugard et al. (2025) [29]	N.R.	Either medial or lateral parapatellar trans-quadricipital	N.R.	N.R.	Yes	N.R.
Sarrami et al. (2025) [37]	N.R.	N.R.	N.R.	N.R.	N.R.	N.R.
Taylor et al. (2025) [41]	N.R.	N.R.	N.R.	N.R.	N.R.	N.R.
Lombardi et al. (2009) [27]	N.R.	Medial parapatellar	**TKA:** yes<br>**UKA:** N.R.	**TKA**: N.R.<br>**UKA:** yes	N.R.	**TKA**: N.R.<br>**UKA:** yes
Driesman et al. (2024) [12]	N.R.	N.R.	N.R.	N.R.	N.R.	N.R.
Lawrence et al. (2024) [24]	N.R.	N.R.	N.R.	N.R.	N.R.	N.R.

Chang et al. (2014) [3]	N.R.	N.R.	N.R.	N.R.	N.R.	N.R.
Fabiano et al. (2024) [13]	N.R.	N.R.	N.R.	N.R.	N.R.	N.R.
Streck et al. (2023) [40]	N.R.	N.R.	N.R.	**TKA**: 88 were cruciate-retaining	N.R.	**UKA**: 14 were mobile-bearing
D'Ambrosi et al. (2021) [8]	N.R.	Medial parapatellar	N.R.	N.R.	Yes	Yes
D'Ambrosi et al. (2023) [7]	No	Medial para-patellar	No	109 were cruciate-retaining	Yes	N.R.
D'Ambrosi et al. (2022) [6]	N.R.	N.R.	N.R.	N.R.	Yes	Yes
Long et al. (2014) [28]	N.R.	N.R.	Yes	Yes	yes (n=34)<br>no (n=2)	N.R.

Legend: N.R. Not reported; **UKA** unicompartmental knee arthroplasty; **TKA** total knee arthroplasty; **OA** Osteoarthritis; **BKA** bicompartmental knee arthroplasty.

Supplement 9 - Return to PA or sports after knee arthroplasty.

First author, year	Type of arthroplasty, patients	Time of pre-operative sports assessment	Baseline sport participation number	Follow-up sport participation number	Definition of return to PA or sports	Return to PA or sports (%)	Time to return to PA or sports (months)
Pioger et al. (2020) [35]	TKA (n=143)	Before surgery	143	142	Until completion of a full 18-hole golf course	142 (99%)	3.7*
Schneider et al. 2020) [39]	TKA (n=46)<br>UKA (n=46)	Sports levels within 5 years before surgery	92	92	Return to level of performance and impact	**Total sample:**<br>*Return to level of impact:*<br>Lower: 42 (36.5%)<br>Similar: 54 (46.9%)<br>Higher: 19 (16.5%)<br><br>*Return to level of performance:*<br>Lower :29 (25.2%)<br>Similar: 30 (26.1%)<br>Higher: 56 (48.7%)<br><br>**UKA:**<br>*Return to level of impact:*<br>Lower: 11 (23.8%)<br>Similar: 27 (58.7%)<br>Higher: 8 (17.4%)<br><br>*Return to level of performance:*<br>Lower: 9 (19.6%)<br>Similar: 12 (26.1%)<br>Higher: 25 (54.4%)<br><br>**TKA:**	N.R.

						Return to level of impact:<br>Lower: 21 (45.7%)<br>Similar: 18 (39.1%)<br>Higher: 7 (15.2%)<br><br>*Return to level of performance:*<br>Lower: 13 (28.3%)<br>Similar: 14 (30.4%)<br>Higher: 19 (41.3%)	
Plassard et al. (2020) [36]	TKA (n=443)	Before surgery	376	376	Returning to sports	376 (100%)	5 (1–36)
Meena et al. (2023) [31]	TKA (n=287)<br>UKA (n=69)	4 weeks before surgery	321	330	Returning to sports	321 (100%)	N.R.
Naylor et al. (2019) [32]	TKA (n=718)	1 year before surgery	293	435	N.R.	N.R.	N.R.
Hopper et al. (2008) [17]	TKA (n=76)<br>UKA (n=34)	Before surgery	**Total**: 85<br>**TKA:** 55<br>**UKA**: 30	**Total:** 64<br>**TKA:** 35<br>**UKA:** 29	Returning to sports Ɨ	**TKA:** 35 (63.6%)<br>**UKA:** 29 (96.7%)	**TKA**: 4.1<br>**UKA:** 3.6
Walton et al. (2006) [46]	TKA (n=120)<br>UKA (n=150)	Before surgery	**Total:** 217<br>**TKA:** 98<br>**UKA**: 119	**Total:** 234<br>**TKA:** 105<br>**UKA:** 129	Returning to sports	**TKA:** 98 (100%)<br>**UKA:** 119 (100%)	**TKA:**<br>Swimming: 13.4 ± 7.8 (4–24)<br>Golf: 12.4 ± 9 (6–18)<br>Bowls: 17.8 ± 14.2 (6–52)<br>Cycling: 12.5 ± 9 (2–24)<br>Tennis: 30 ± 31.1 (6–52)<br><br>**UKA:**<br>Swimming: 9.4 ± 7.7 (2–26)<br>Golf: 13 ± 8.7 (3–32)<br>Bowls: 24.6 ± 24.7 (4–104)<br>Cycling: 11.8 ± 11.0 (1–36)

							Tennis: 10.1 ± 2.5 (8–13)
Tramer et al. (2022) [42]	TKA (n=54)	Before surgery	54	44	Return to golfing activity	44 (81.5%)	5.3 ± 3.1
Jassim et al. (2019) [21]	TKA (n=24)	Before surgery	24	24	Returning to sports	24 (100%)	N.R.
Cozzarelli et al. (2024) [4]	Medial UKA (n=278) Non-specific UKA (n=18)	5 years before surgery	256	209	Returning to sports	209 (81.6%)	N.R.
Ho et al. (2015) [16]	TKA (n=39) UKA (n=33)	Before the onset of knee symptoms	**TKA:** 30 **UKA:** 30	**TKA:** 28 **UKA:** 26	Returning to sports	**TKA:** 25 (83%) **UKA:** 26 (87%)	**Total:** 3 [2–6] 1–62 # **TKA**: 4 [2–12] 1–62 # **UKA**: 2 [1.5–4.9] 1–12 #
Carlo et al. (2022) [2]	Medial UKA (n=137)	Before surgery	137	121	Returning to sports	120 (87.7%)	6 (5-7)
Lo Presti et al. (2019) [26]	Medial UKA (n=53)	Before the onset of restricting symptoms (no more than 1 year before surgery)	53	48	Returning to sports	48 (90.6%)	≤6: 19 patients >6: 29 patients
Legnani et al. (2023) [25]	UKA (n=38)	Before surgery	38	35	Returning to sports	35 (92%)	≤6: 28 patients >6: 7 patients
Walker et al. (2015) [45]	Lateral UKA (n=45)	Before the onset of the first restricting symptoms	42	43	Returning to sports	41 (98%)	≤6: 35 patients >6: 6 patients

Kleeblad et al. (2020) [23]	UKA (n=164)	Sports levels within 5 years before surgery	132	147	Returning to sports	125 (94.7%)	4.4 ± 3.5 (0.3–24.0)
Ventura et al. (2022) [44]	TKA (n=97)	Before surgery	64	52	Returning to sports **Ɨ**	52 (81%)	N.R.
Pietschmann et al. (2013) [34]	Medial UKA (n=131)	Before surgery	78	69	Returning to sports	63 (80.1%)	N.R.
Zimmerer et al. (2021) [49]	Lateral UKA (n=19)	Before the onset of knee symptoms	15	13	Returning to sports	13 (86%)	≤6: 9 patients<br>>6: 4 patients
Hepperger et al. (2018) [15]	TKA (n=200)	Before surgery	124	140	N.R.	N.R.	N.R.
Kim et al. (2019) [22]	UKA (n=42)	Before the onset of knee symptoms	34	32	Returning to sports	32 (94.1%)	3.8
Yim et al. (2013) [48]	UKA (n=50)	Before surgery	42	30	Returning to sports	30 (71.4%)	N.R.
Yamagishi et al. (2006) [47]	TKA (n=11)	Before surgery	11	11	Returning to sports	11 (100%)	N.R.
Huch et al. (2005) [19]	TKA (n=389)	Before surgery	163	131	N.R.	N.R.	N.R.
Jahnke et al. (2015) [20]	Medial UKA (n=135)	Before surgery	100	114	N.R.	N.R.	N.R.
Deng et al. (2023) [11]	BKA (n=25)<br>TKA (n=50)	Before surgery	N.R.	N.R.	Returning to sports	**BKA**: N.R. (71.6%)<br>**TKA**: N.R. (56.4%)	N.R.
Daffara et al. (2024) [10]	UKA (n=136)	Before surgery	27	33	Returning to sports	N.R. (93.1%)	N.R.

Hariri et al. (2023) [14]	UKA (n=37)	Before the onset of the first restricting symptoms	32	30	Returning to sports	28 (87.5%)	N.R.
Schmidt et al. (2023) [38]	UKA (n=92)	Before the onset of the first restricting symptoms	92	N.R.	Returning to sports	N.R.	5.5 ± 3.4
Antonelli et al. (2023) [1]	TKA (n= 2288)<br>UKA (n= 189)	Before surgery	**TKA**: 218<br>**UKA**: 38	**TKA**: 22<br>**UKA**: 7	Returning to running	**TKA**: 9 (4.1%)<br>**UKA**: 7 (18.4%)	N.R.
Maugard et al. (2025) [29]	TKA (n=110)	Before surgery	N.R.	N.R.	Returning to physical activity	N.R. (72.7%)	6.3 ± 5.2
Sarrami et al. (2025) [37]	TKA (n=257)	12 months before surgery	N.R.	N.R.	Returning to sports	N.R. (98.0%)	N.R.
Taylor et al. (2025) [41]	TKA (n=326)<br>UKA (n=56)	24 months before surgery	**TKA**: 88<br>**UKA**: 6	**TKA**: 16<br>**UKA**: 2	Return to playing racket sports	**TKA**: 16 (18.0%)<br>**UKA**: 2 (33.0%)	N.R.
Lombardi et al. (2009) [27]	TKA (n=115)<br>UKA (n=115)	Before surgery	N.R.	N.R.	Returning to sports	N.R.	2.8 ± 2.8
Driesman et al. (2024) [12]	TKA (n=332)	Before surgery	199	131	Returning to cycling	120 (60.3%)	N.R.
Lawrence et al. (2024) [24]	TKA (n=784)	Two years preoperatively	535	454	Returning to sports	252 (47.2%)	N.R.
Chang et al. (2014) [3]	TKA (n=369)	Before surgery	N.R.	N.R.	N.R.	N.R.	N.R.
Fabiano et al. (2024)	TKA (n=83)	Before surgery	N.R.	N.R.	N.R.	N.R.	N.R.

[13]							
Streck et al. (2023) [40]	TKA (n=1746) UKA (n=161)	Before surgery	N.R.	N.R.	N.R.	N.R.	N.R.
D'Ambrosi et al. (2022) [9]	Medial UKA (n=73) Lateral UKA (n=12)	Before surgery	N.R.	N.R.	N.R.	N.R.	N.R.
D'Ambrosi et al. (2021) [8]	UKA (n=41)	Before surgery	N.R.	N.R.	N.R.	N.R.	N.R.
D'Ambrosi et al. (2023) [7]	TKA (n=227)	Before surgery	227	224	Returning to sports	224 (98.7%)	N.R.
D'Ambrosi et al. (2022) [6]	UKA (n=172)	Day before surgery	N.R.	N.R.	N.R.	N.R.	N.R.
Long et al. (2014) [28]	TKA (n=36)	Before surgery	N.R.	N.R.	N.R.	N.R.	N.R.

Legend: N.R. Not reported; **RTS** Return to sport**; UKA** unicompartmental knee arthroplasty**; TKA** total knee arthroplasty; **BKA** bicompartmental knee arthroplasty

*Median.

Ɨ It is unclear whether the RTS refers only to the participants who played sports before surgery.

Median [interquartile] range.

Supplement 10 - Sensitivity analysis for return to PA or sports proportion, excluding studies with ambiguous assessment of return to PA or sports.

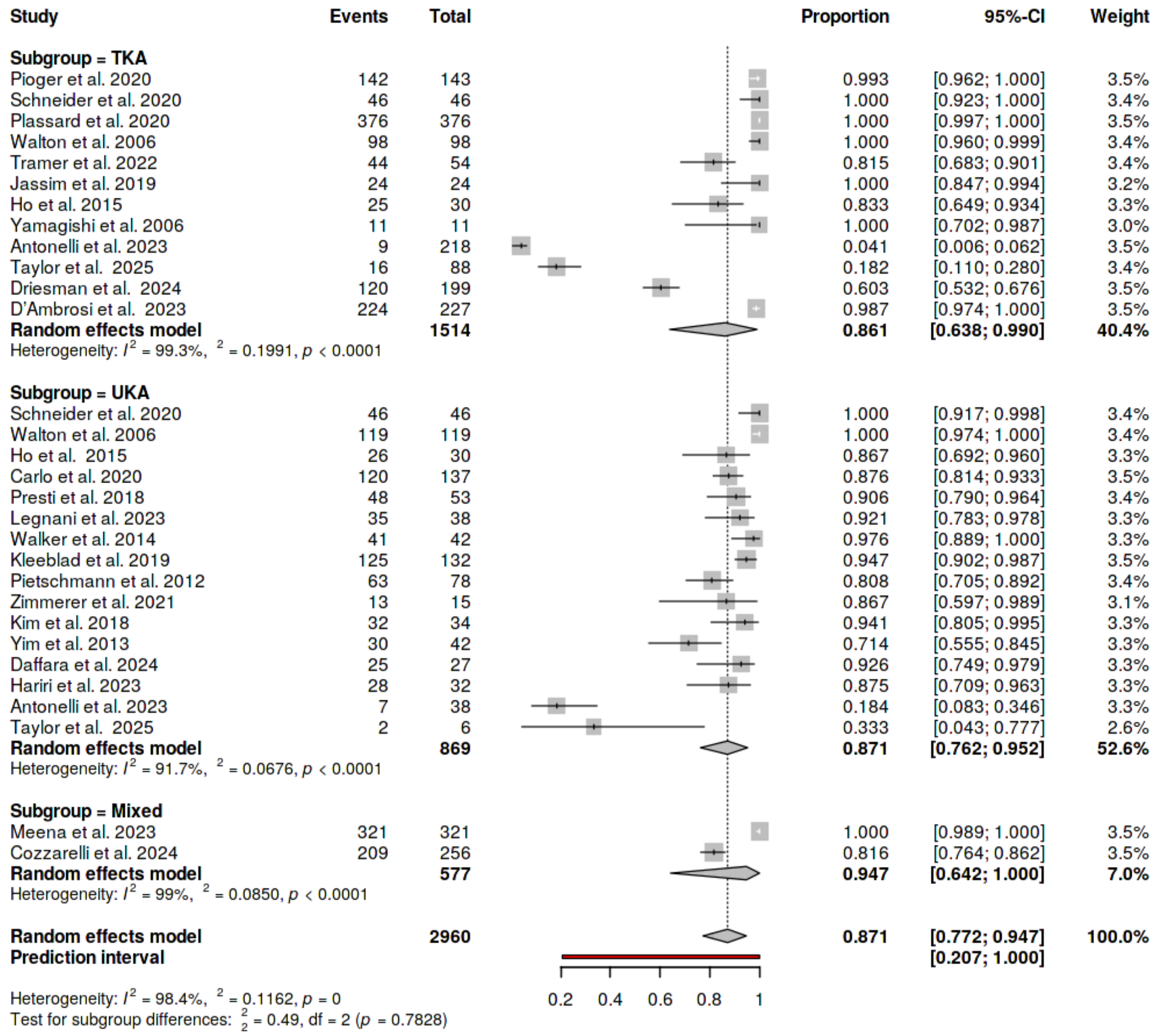

Supplement 11 - Funnel plot to assess the risk of publication bias for return to PA or sports proportion.

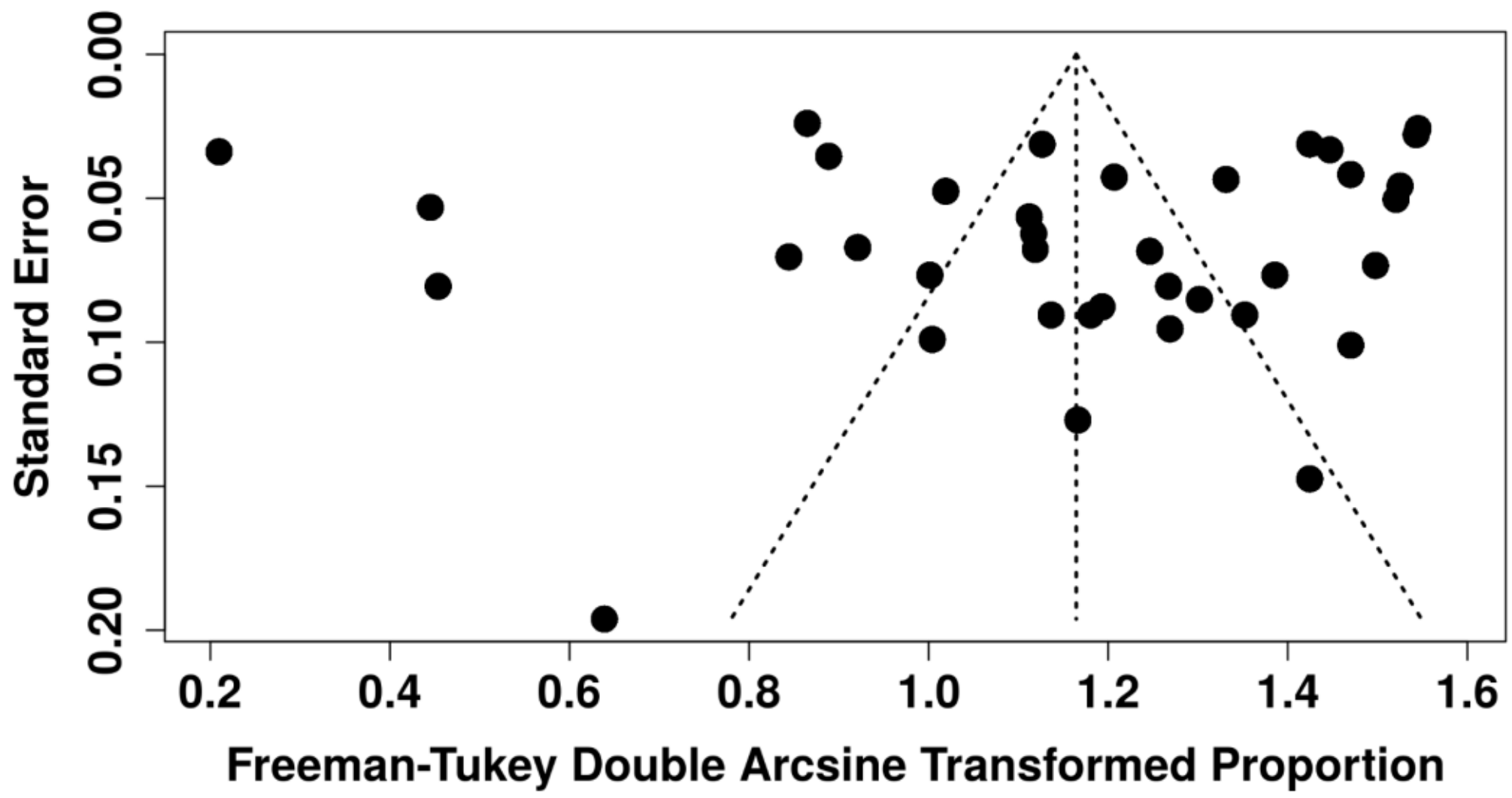

Supplement 12 - Sensitivity analysis between-studies odds ratio (OR) of return to PA or sports proportion, excluding studies with ambiguous assessment of return to PA or sports.

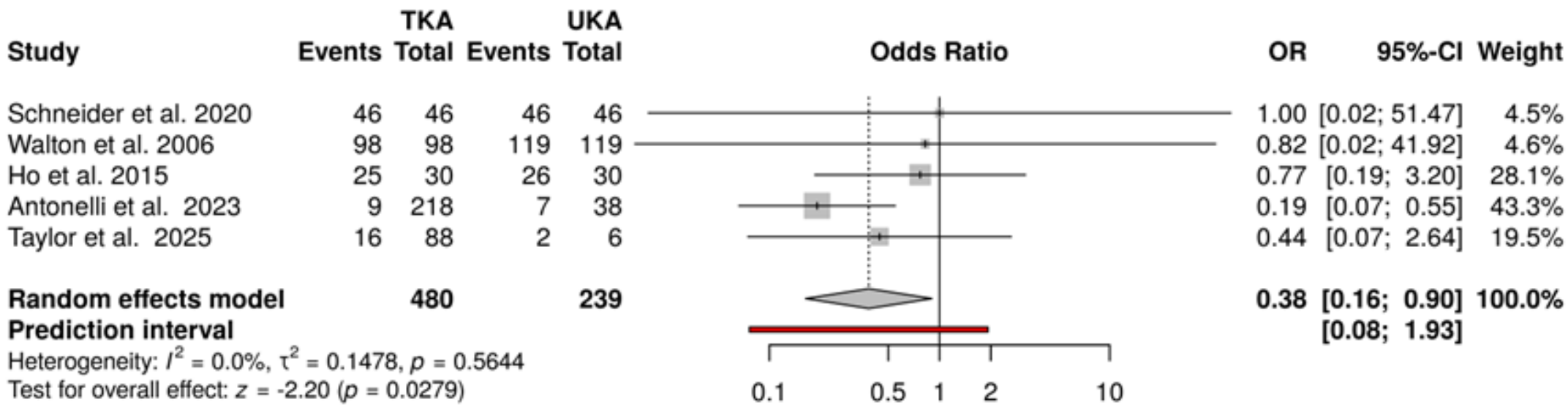

Supplement 13 - Funnel plot to assess the risk of publication bias for pre-to-post Tegner score.

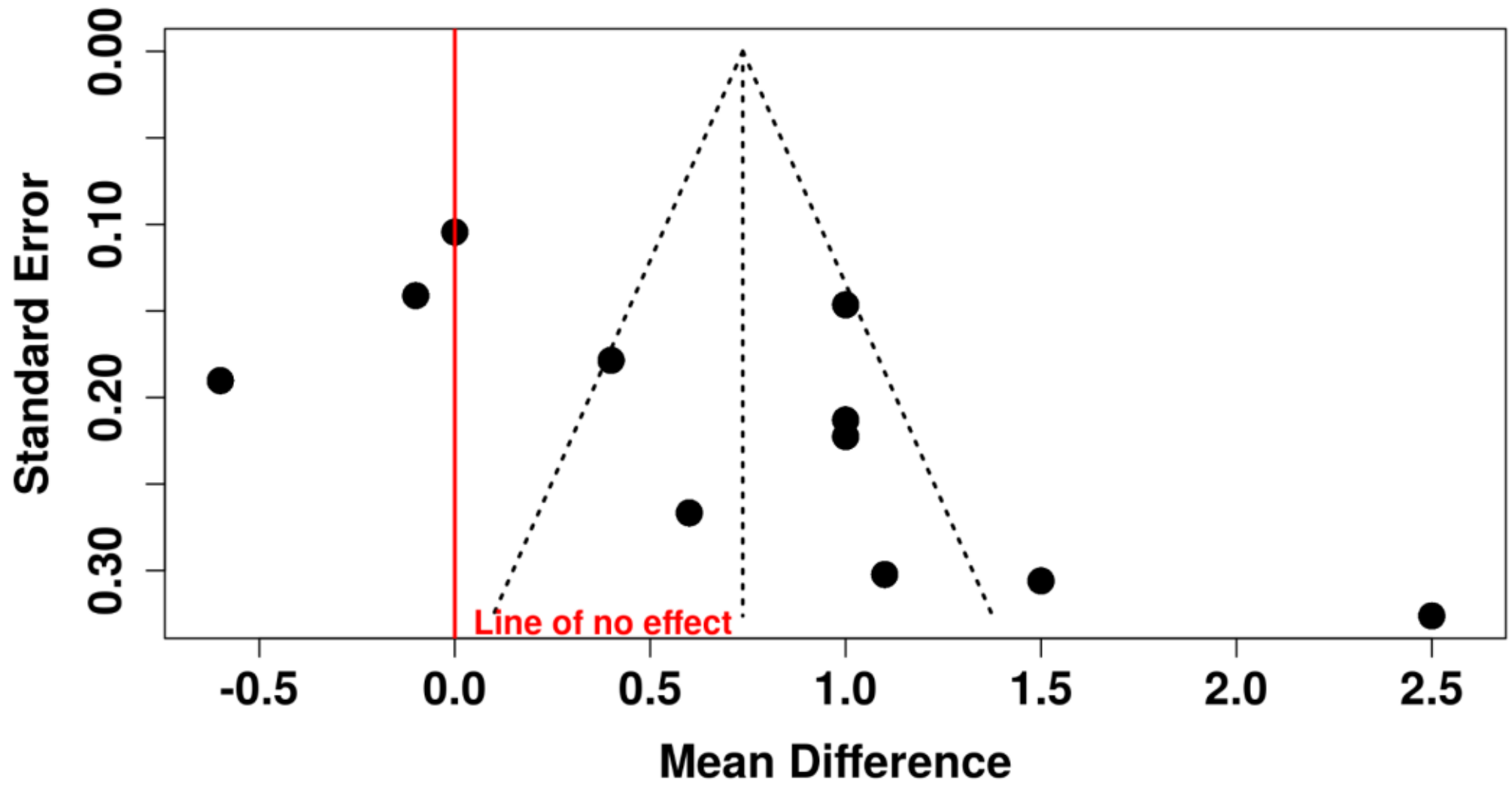

Supplement 14 - Sensitivity analysis for pre-to-post UCLA score, excluding studies with pre-operative non-active participants.

Study	Follow-up Total	Follow-up Mean	Follow-up SD	Baseline Total	Baseline Mean	Baseline SD	MD	95%-CI	Weight
**subgroup = TKA**									
Pioger et al. 2020	143	7.80	1.1000	143	7.00	2.0000	0.80	[0.43; 1.17]	12.7%
Schneider et al. 2020	46	6.62	1.9000	46	5.30	2.4000	1.32	[0.44; 2.20]	12.0%
Jassim et al. 2019	24	7.21	0.9000	24	7.50	1.1000	-0.29	[-0.86; 0.28]	12.5%
D'Ambrosi et al. 2023	227	7.00	1.0000	227	4.80	1.0000	2.20	[2.02; 2.38]	12.8%
**Random effects model**	**440**			**440**			**1.02**	**[-0.01; 2.04]**	**50.0%**
Heterogeneity: I^2 = 96.9%, ^{2} = 1.0168, p < 0.0001									
**subgroup = UKA**									
Schneider et al. 2020	46	6.90	2.0000	46	5.90	2.5000	1.00	[0.07; 1.93]	12.0%
Carlo et al. 2020	71	7.30	1.3000	71	4.40	1.3000	2.90	[2.47; 3.33]	12.6%
Schmidt et al. 2023	92	7.20	2.0000	92	9.90	0.3000	-2.70	[-3.11; -2.29]	12.6%
D'Ambrosi et al. 2022	172	6.30	0.7000	172	3.80	1.1000	2.50	[2.31; 2.69]	12.8%
**Random effects model**	**381**			**381**			**0.93**	**[-1.58; 3.43]**	**50.0%**
Heterogeneity: I^2 = 99.4%, ^{2} = 6.4553, p < 0.0001									
**Random effects model**	**821**			**821**			**0.97**	**[-0.29; 2.23]**	**100.0%**
**Prediction interval**								**[-3.53; 5.47]**	

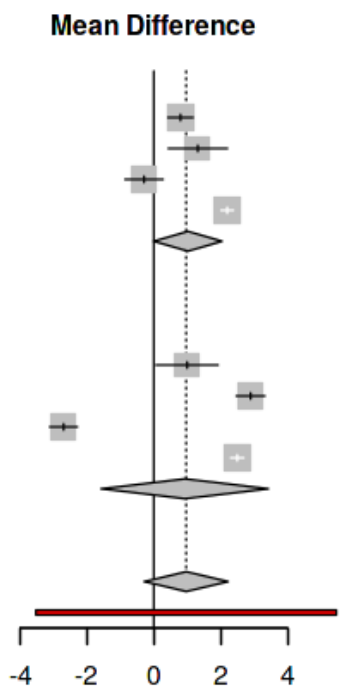

Heterogeneity: I^2 = 98.9%, ^{2} = 3.2075, p < 0.0001
Test for overall effect: z = 1.51 (p = 0.1305)
Test for subgroup differences: $^2_1$ = 0.00, df = 1 (p = 0.9463)

Supplement 15 - Sensitivity analysis for pre-to-post UCLA score, excluding studies with estimated central tendency and variance data.

Study	Follow-up Total	Follow-up Mean	Follow-up SD	Baseline Total	Baseline Mean	Baseline SD	MD	95%-CI	Weight
**subgroup = TKA**									
Pioger et al. 2020	143	7.80	1.1000	143	7.00	2.0000	0.80	[0.43; 1.17]	6.1%
Schneider et al. 2020	46	6.62	1.9000	46	5.30	2.4000	1.32	[0.44; 2.20]	5.6%
Jassim et al. 2019	24	7.21	0.9000	24	7.50	1.1000	-0.29	[-0.86; 0.28]	5.9%
Ho et al. 2015	39	6.10	1.4000	39	7.20	1.6000	-1.10	[-1.77; -0.43]	5.8%
Maugard et al. 2025	110	5.90	2.0000	110	4.40	1.8000	1.50	[1.00; 2.00]	6.0%
Chang et al. 2014	369	4.80	1.4000	369	4.50	1.8000	0.30	[0.07; 0.53]	6.1%
Fabiano et al. 2024	83	4.60	0.3000	83	3.90	0.2000	0.70	[0.62; 0.78]	6.2%
**Random effects model**	**814**			**814**			**0.46**	**[-0.20; 1.12]**	**41.6%**
Heterogeneity: I^2 = 90.2%, ^{2} = 0.7262, p < 0.0001									
**subgroup = UKA**									
Schneider et al. 2020	46	6.90	2.0000	46	5.90	2.5000	1.00	[0.07; 1.93]	5.5%
Ho et al. 2015	33	7.40	1.6000	33	8.10	1.5000	-0.70	[-1.45; 0.05]	5.7%
Walker et al. 2014	45	6.70	1.5000	45	5.30	2.3000	1.40	[0.60; 2.20]	5.7%
Zimmerer et al. 2021	19	6.40	1.3000	19	4.10	1.7000	2.30	[1.34; 3.26]	5.5%
Jahnke et al. 2014	135	6.30	1.1000	135	6.10	1.3000	0.20	[-0.09; 0.49]	6.1%
Hariri et al. 2023	37	6.00	1.5000	37	3.60	1.8000	2.40	[1.65; 3.15]	5.7%
Schmidt et al. 2023	92	7.20	2.0000	92	9.90	0.3000	-2.70	[-3.11; -2.29]	6.0%
D'Ambrosi et al. 2021	41	6.10	0.8000	41	3.70	1.2000	2.40	[1.96; 2.84]	6.0%
**Random effects model**	**448**			**448**			**0.77**	**[-0.48; 2.02]**	**46.3%**
Heterogeneity: I^2 = 98%, ^{2} = 3.1353, p < 0.0001									
**subgroup = Mixed**									
Kleeblad et al. 2019	179	6.80	2.0000	179	5.90	2.2000	0.90	[0.46; 1.34]	6.0%
D'Ambrosi et al. 2022	85	6.30	0.6000	85	3.60	1.2000	2.70	[2.41; 2.99]	6.1%
**Random effects model**	**264**			**264**			**1.81**	**[0.06; 3.55]**	**12.1%**
Heterogeneity: I^2 = 97.8%, ^{2} = 1.5524, p < 0.0001									
**Random effects model**	**1526**			**1526**			**0.76**	**[0.09; 1.44]**	**100.0%**
**Prediction interval**								**[-2.26; 3.79]**	

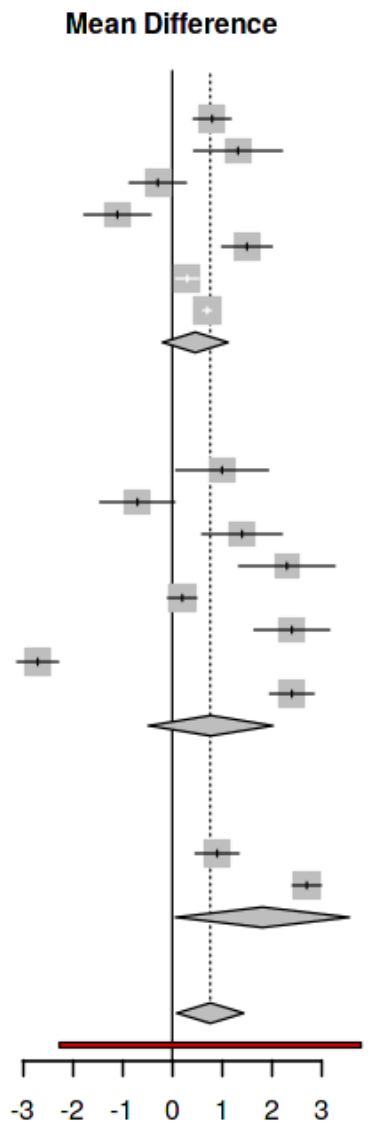

Heterogeneity: I^2 = 97.5%, ^{2} = 1.9194, p < 0.0001
Test for overall effect: z = 2.22 (p = 0.0267)
Test for subgroup differences: $^2_2$ = 2.03, df = 2 (p = 0.3617)

Supplement 16 - Funnel plot to assess risk of publication bias for UCLA scores.

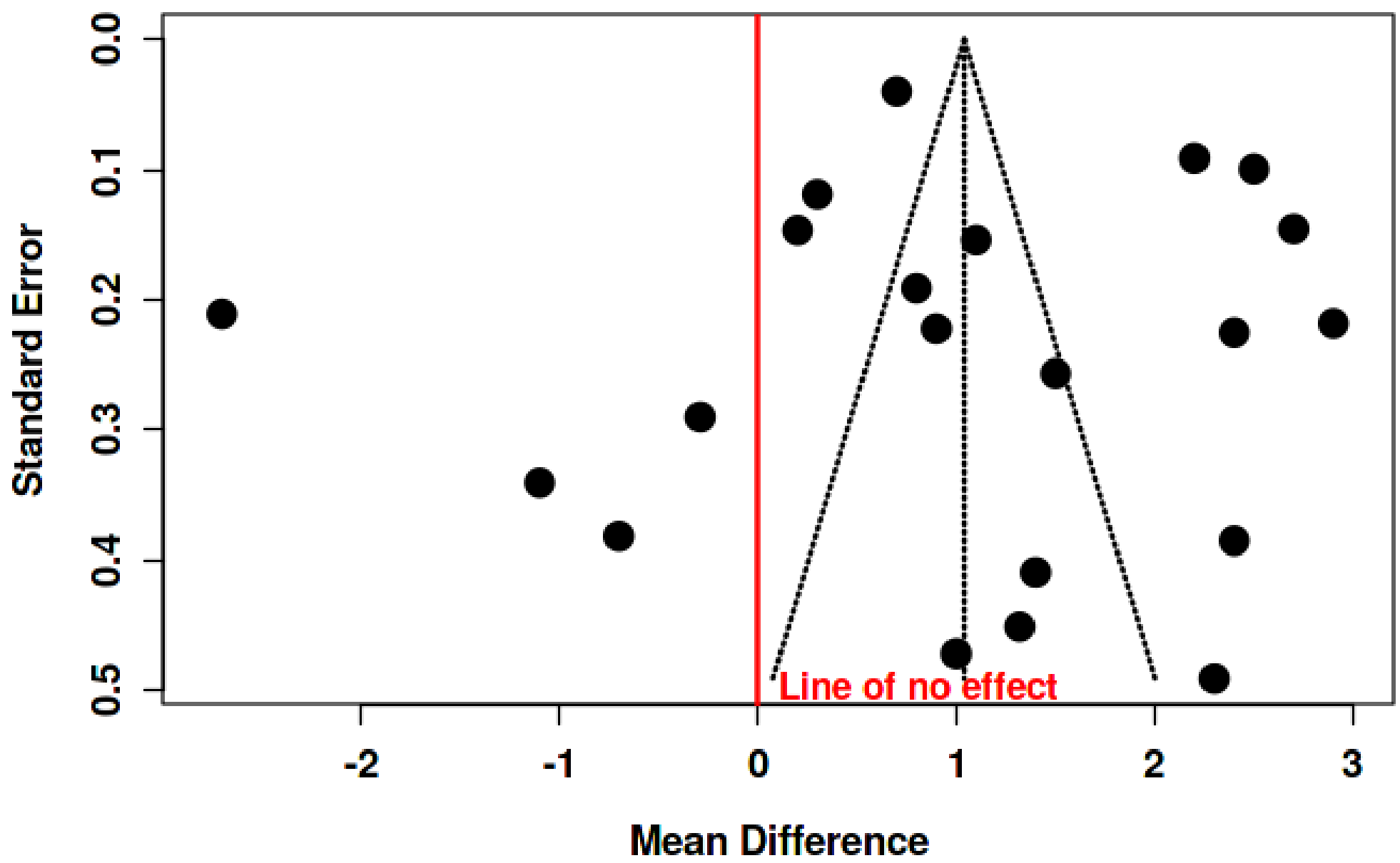

Supplement 17 - Sensitivity analysis for pre-to-post HAAS, excluding studies with pre-operative non-active participants.

Study	Total	Mean	Follow-up SD	Total	Mean	Baseline SD	MD	95%-CI	Weight
**subgroup = UKA**									
Schneider et al. 2020	46	6.90	2.0000	46	5.90	2.5000	1.00	[0.07; 1.93]	24.6%
D'Ambrosi et al. 2022	172	11.00	0.9000	172	4.50	1.5000	6.50	[6.24; 6.76]	25.3%
**Random effects model**	**218**			**218**			**3.77**	**[-1.60; 9.14]**	**50.0%**
Heterogeneity: I^2 = 99.2%, ^{2} = 14.8882, p < 0.0001									
**subgroup = TKA**									
Schneider et al. 2020	46	6.60	1.9000	46	5.30	2.4000	1.30	[0.42; 2.18]	24.7%
D'Ambrosi et al. 2023	227	10.80	1.2000	227	6.30	1.0000	4.50	[4.30; 4.70]	25.4%
**Random effects model**	**273**			**273**			**2.93**	**[-0.17; 6.04]**	**50.0%**
Heterogeneity: I^2 = 97.9%, ^{2} = 4.9142, p < 0.0001									
**Random effects model**	**491**			**491**			**3.36**	**[0.77; 5.94]**	**100.0%**
**Prediction interval**								**[-5.97; 12.68]**	

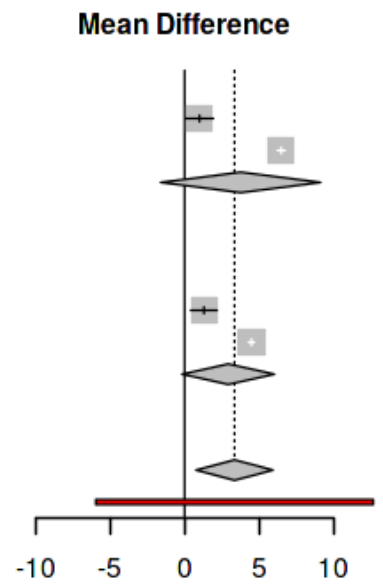

Heterogeneity: I^2 = 99.0%, ^{2} = 6.8501, p < 0.0001
Test for overall effect: z = 2.54 (p = 0.0110)
Test for subgroup differences: χ^2_1 = 0.07, df = 1 (p = 0.7911)

Supplement 18 - Sensitivity analysis for pre-to-post HAAS, excluding studies with estimated central tendency and variance data.

		Follow-up			Baseline				
**Study**	**Total**	**Mean**	**SD**	**Total**	**Mean**	**SD**	**MD**	**95%-CI**	**Weight**
**subgroup = UKA**									
Schneider et al. 2020	46	6.90	2.0000	46	5.90	2.5000	1.00	[0.07; 1.93]	18.9%
Kleeblad et al. 2020	164	11.10	2.8000	164	9.10	3.5000	2.00	[1.31; 2.69]	20.0%
D'Ambrosi et al. 2022	85	6.20	0.6000	85	3.40	1.7000	2.80	[2.42; 3.18]	21.1%
D'Ambrosi et al. 2021	41	10.30	1.3000	41	6.10	0.8000	4.20	[3.73; 4.67]	20.9%
**Random effects model**	**336**			**336**			**2.54**	**[1.23; 3.85]**	**80.9%**
Heterogeneity: I^2 = 94.3%, ^{2} = 1.6730, p < 0.0001									
**subgroup = TKA**									
Schneider et al. 2020	46	6.60	1.9000	46	5.30	2.4000	1.30	[0.42; 2.18]	19.1%
**Random effects model**	**382**			**382**			**2.31**	**[1.18; 3.43]**	**100.0%**
**Prediction interval**								**[-1.48; 6.09]**	

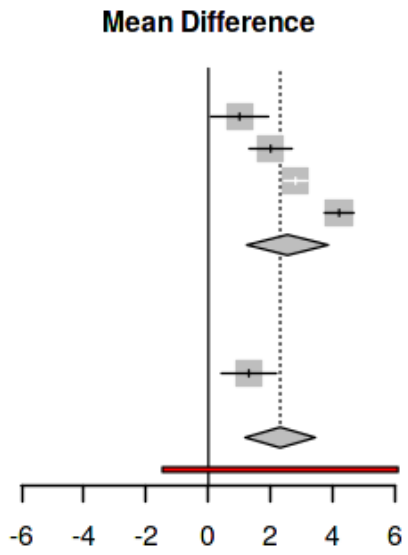

Heterogeneity: I^2 = 93.9%, ^{2} = 1.5251, p < 0.0001
Test for overall effect: z = 4.01 (p < 0.0001)
Test for subgroup differences: $^2_1$ = 2.37, df = 1 (p = 0.1239)

Supplement 19 - PA scores at baseline and follow-up.

First author, year	Type of arthroplasty, patients	Outcome	Pre-operative	Post-operative
Pioger et al. (2020) [35]	TKA (n=143)	UCLA	7.0 ± 2.0	7.8 ± 1.1
Schneider et al. (2020) [39]	TKA (n=46)<br>UKA (n=46)	UCLA	**Total sample:** 5.5 ± 2.4<br>**UKA:** 5.9 ± 2.5<br>**TKA**: 5.3 ± 2.4	**Total sample:** 6.7 ± 2.0<br>**UKA:** 6.9 ± 2.0<br>**TKA:** 6.6 ± 1.9
		HAAS	**Total sample:** 8.4 ± 3.8<br>**UKA:** 9.9 ± 3.8<br>**TKA:** 7.1 ± 3.4	**Total sample:** 10.9 ± 3.6<br>**UKA**: 12.4 ± 3.8<br>**TKA**: 9.5 ± 3.4
Plassard et al. (2020) [36]	TKA (n=443)	UCLA	4.4	5.9
Meena et al. (2023) [31]	TKA (n=287)<br>UKA (n=69)	Tegner †	**Total:** 3 (2–5)<br>**TKA:** 3 (2–5)<br>**UKA:** 3 (3–4)	**Total:** 3 (3–5)<br>**TKA:** 3 (3–5)<br>**UKA:** 4 (3–6)
Walton et al. (2006) [46]	TKA (n=120)<br>UKA (n=150)	Grimby score	N.R.	**TKA:** 2.8 ± 1.1 (1-6)<br>**UKA:** 3.9 ± 1.3 (1-6)
Jassim et al. (2019) [21]	TKA (n=24)	UCLA	7.5 ± 1.1 (6-10)	7.29 ± 0.9 (6-9)
Ho et al. (2015) [16]	TKA (n=39)<br>UKA (n=33)	UCLA	**Total:** 7.7 ± 1.6 (4–10)<br>**TKA:** 7.2 ± 1.6 (4–10)<br>**UKA:** 8.1 ± 1.5 (6–10)	**Total:** 6.7 ± 1.6 (3-10)<br>**TKA:** 6.1 ± 1.4<br>**UKA:**7.4 ± 1.6
Carlo et al. (2022) [2]	Medial UKA (n=137)	UCLA OA < grade III	4.5 ± 1.0	7.0 ± 1.5
		UCLA OA ≥ grade III	4.4 ± 1.6	7.7 ± 1.0
Legnani et al. (2023) [25]	UKA (n=42)	Tegner	3.5 (1–6)	4.5 (1–8)
Walker et al. (2015) [45]	Lateral UKA (n=45)	UCLA	5.3 ± 2.3	6.7 ± 1.5
		Tegner	2.9 ± 1.6	3.5 ± 0.8
Kleeblad et al. (2020) [23]	UKA (n=164)	HAAS	9.1 ± 3.5	11.1 ± 2.8
		UCLA	6.0 ± 2.2	6.8 ± 2.0
Ventura et al. (2022) [44]	TKA (n=97)	Tegner †	3 (1–6)	4 (1–8)
Zimmerer et al. (2021) [49]	Lateral UKA (n=19)	UCLA	4.1 ± 1.7 (2.0–9.0)	6.4 ± 1.3 (4.0–9.0)

Hepperger et al. (2018) [15]	TKA (n=200)	Tegner †	3.0 (2.0 to 4.0)	3.0 (3.0 to 4.0)
Kim et al. (2019) [22]	UKA (n=42)	UCLA	3.1	5.2
		Tegner	2.1	3.6
Yim et al. (2013) [48]	UKA (n=50)	Tegner	3.2 ± 0.9	2.6 ± 0.9
Yamagishi et al. (2006) [47]	TKA (n=11)	Tegner	1.5 (1-3)	4.0 (3-6)
Jahnke et al. (2015) [20]	Medial UKA (n=135)	Tegner	4.1 ± 1.3	4.0 ± 1.0
		HAS	23.8 ± 18.5	29.3 ± 18.5
		UCLA	6.1 ± 1.3	6.3 ± 1.1
Daffara et al. (2024) [10]	UKA (n=136)	UCLA ‡	N.R.	5.9 ± 1.2
D'Ambrosi et al. (2022) [9]	Medial UKA (n=73) Lateral UKA (n=12)	UCLA	3.6 ± 1.2	6.3 ± 0.6
		HAAS	3.4 ± 1.7	6.2 ± 0.6
Hariri et al. (2023) [14]	UKA (n=37)	Tegner	2.2 ± 1.3	3.3 ± 1.3
		UCLA	3.6 ± 1.8	6.0 + 1.5
Schmidt et al. (2023) [38]	UKA (n=92)	UCLA	9.9 ± 0.3	7.2 ± 2.0
Maugard et al. (2025) [29]	TKA (n=110)	UCLA	4.4 ± 1.8	5.9 ± 2.0
Sarrami et al. (2025) [37]	TKA (n=257)	UCLA	5.3 ± 2.3 #	6.4 ± 0.9 #
Lombardi et al. (2009) [27]	TKA (n=115) UKA (n=115)	LEAS	N.R.	**TKA:** 9.5 ± 2.4 **UKA:** 11.5 ± 2.8
Chang et al. (2014) [3]	TKA (n=369)	UCLA	4.5 ± 1.8	4.8 ± 1.4
Fabiano et al. (2024) [13]	TKA (n=83)	UCLA	3.9 (3.5–4.1)	4.6 (4.3–5.0)
Streck et al. (2023) [40]	TKA (n=1746) UKA (n=161)	LEAS	**TKA:** 10.0 ± 3.2 **UKA:** 10.2 ± 3.1	**TKA:** 11.1 ± 3.5 **UKA:** 11.9 ± 3.6
D'Ambrosi et al. (2021) [8]	UKA (n=41)	UCLA	3.7 ± 1.2	6.1 ± 0.8
		HAAS	6.1 ± 0.8	10.3 ± 1.3
D'Ambrosi et al. (2023) [7]	TKA (n=227)	UCLA ‡	4.8 ± 1.0	7.0 ± 1,0
		HAAS ‡	6.3 ± 1.0	10.8 ± 1.2
D'Ambrosi et al. (2021) [6]	UKA (n=172)	UCLA ‡	3.8 ± 1.1	6.3 ± 0.7
		HAAS ‡	4.5 ± 1.5	11.0 ± 0.9
Long et al. (2014) [28]	TKA (n=36)	Tegner	1.5 ± 0.9	3.0 ± 1.6

Legend: HAAS High-Activity Arthroplasty Score; **HAS** Heidelberg Sports Activity Score; **UCLA** University of California, Los Angeles Activity Score; **N.R**. Not reported; **UKA** unicompartmental knee arthroplasty**; TKA** total knee arthroplasty; **OA** Osteoarthritis; **LEAS** Lower extremity activity scale

* Takes into consideration 23 patellofemoral arthroplasty patients.

† Median (IQR, 25th–75th percentile).

# Standard deviations estimated based on mean difference and P value.

‡ Combines TKA/UKA subgroups.

List of references

1. Antonelli, B, R Teng, R G Breslow, M Jamison, M Hepinstall, R Schwarzkopf, W E Moschetti and A F Chen (2023) Few runners return to running after total joint arthroplasty, while others initiate running. JAAOS Global Research & Reviews 7(4): e23.

2. Carlo, M, et al. (2022) Return to sports after medial unicompartmental knee arthroplasty in patients with concomitant patella-femoral osteoarthritis: multicenter retrospective cohort study with minimum 5-year follow-up. Eur J Orthop Surg Traumatol 32(1): 55-61. https://doi.org/10.1007/s00590-021-02925-1

3. Chang, M J, S H Kim, Y G Kang, C B Chang and T K Kim (2014) Activity levels and participation in physical activities by Korean patients following total knee arthroplasty. BMC Musculoskelet Disord 15: 240. 10.1186/1471-2474-15-240

4. Cozzarelli, N F, I A Khan, A Arshi, M B Sherman, J H Lonner and Y A Fillingham (2024) Return to Sport After Unicompartmental Knee Arthroplasty and Patello-Femoral Arthroplasty. J Arthroplasty 39(8): 1988-1995.e1985. https://doi.org/10.1016/j.arth.2024.02.004

5. Cumpston, M, T Li, M J Page, J Chandler, V A Welch, J P Higgins and J Thomas (2019) Updated guidance for trusted systematic reviews: a new edition of the Cochrane Handbook for Systematic Reviews of Interventions. Cochrane Database Syst Rev 10(10): Ed000142. https://doi.org/10.1002/14651858.Ed000142

6. D'Ambrosi, R, F M Anghilieri, K Corona, I Mariani, F Valli, N Ursino and M T Hirschmann (2022) Similar rates of return to sports and BMI reduction regardless of age, gender and preoperative BMI as seen in matched cohort of hypoallergenic and standard Cobalt Chromium medial unicompartmental knee arthroplasty. Knee Surg Sports Traumatol Arthrosc 30(3): 890-898. 10.1007/s00167-021-06467-1

7. D'Ambrosi, R, L Mangiavini, R Loucas, M Loucas, A Brivio, I Mariani, N Ursino and F Migliorini (2023) Similar rate of return to sports activity between posterior-stabilised and cruciate-retaining primary total knee arthroplasty in young and active patient. Knee Surg Sports Traumatol Arthrosc 31(2): 551-558. 10.1007/s00167-022-07176-z

8. D'Ambrosi, R, A Nuara, I Mariani, K Corona, S Mogos, F Catellani, M Hantes and N Ursino (2021) Return to physical activity and change in body mass index after hypoallergenic medial mobile-bearing unicompartmental knee arthroplasty. J Orthop Traumatol 22(1): 36. 10.1186/s10195-021-00598-4

9. D'Ambrosi, R, C Ursino, I Mariani, K Corona, J Dahmen, F V Sciarretta, F Valli and N Ursino (2022) No difference in return to amateur sports after medial and lateral unicompartmental knee

arthroplasty in patients younger than 65 years. Knee Surg Sports Traumatol Arthrosc 30(3): 1050-1056. 10.1007/s00167-021-06526-7

10. Daffara, V, F Zambianchi, E Festa, R Cuoghi Costantini, S Clemenza and F Catani (2024) High return to sport rate in patients undergoing image-based robotic arm assisted unicompartmental knee arthroplasty. Arch Orthop Trauma Surg 144(9): 4037-4044. 10.1007/s00402-023-05141-0

11. Deng, W, H Shao, H Tang, Q Tang, Z Wang, D Yang and Y Zhou (2023) Better PROMs and higher return-to-sport rate after modular bicompartmental knee arthroplasty than after total knee arthroplasty for medial and patellofemoral compartment osteoarthritis. Front Surg 9: 1078866. 10.3389/fsurg.2022.1078866

12. Driesman, A S, R M Johnson, C C Yang, T M Miner, D A Dennis and J M Jennings (2024) Return to Cycling After Total Joint Arthroplasty. J Arthroplasty 39(11): 2686-2691.e2682. 10.1016/j.arth.2024.05.041

13. Fabiano, G, T O Smith, S Parsons, A Ooms, S Dutton, B Fordham, C Hing, S Lamb and R Pinedo-Villanueva (2024) Physical activity and health-related quality of life of patients with chronic knee pain after total knee replacement: Analysis of the PEP-TALK trial. Knee 46: 80-88. 10.1016/j.knee.2023.11.012

14. Hariri, M, M Hagemann, P Mick, J Deisenhofer, B Panzram, M Innmann, T Reiner, T Renkawitz and T Walker (2023) Physical Activity of Young Patients following Minimally Invasive Lateral Unicompartmental Knee Replacement. J Clin Med 12(2). 10.3390/jcm12020635

15. Hepperger, C, P Gföller, E Abermann, C Hoser, H Ulmer, E Herbst and C Fink (2018) Sports activity is maintained or increased following total knee arthroplasty. Knee Surg Sports Traumatol Arthrosc 26(5): 1515-1523. https://doi.org/10.1007/s00167-017-4529-3

16. Ho, J C, R N Stitzlein, C J Green, T Stoner and M I Froimson (2016) Return to Sports Activity following UKA and TKA. J Knee Surg 29(3): 254-259. https://doi.org/10.1055/s-0035-1551835

17. Hopper, G P and W J Leach (2008) Participation in sporting activities following knee replacement: total versus unicompartmental. Knee Surg Sports Traumatol Arthrosc 16(10): 973-979. https://doi.org/10.1007/s00167-008-0596-9

18. Hozo, S P, B Djulbegovic and I Hozo (2005) Estimating the mean and variance from the median, range, and the size of a sample. BMC Med Res Methodol 5: 13. https://doi.org/10.1186/1471-2288-5-13

19. Huch, K, K A Müller, T Stürmer, H Brenner, W Puhl and K P Günther (2005) Sports activities 5 years after total knee or hip arthroplasty: the Ulm Osteoarthritis Study. Ann Rheum Dis 64(12): 1715-1720. https://doi.org/10.1136/ard.2004.033266

20. Jahnke, A, J K Mende, G S Maier, G A Ahmed, B A Ishaque, H Schmitt, M Rickert, M Clarius and J B Seeger (2015) Sports activities before and after medial unicompartmental knee arthroplasty using the new Heidelberg Sports Activity Score. Int Orthop 39(3): 449-454. https://doi.org/10.1007/s00264-014-2524-6

21. Jassim, S S, J Tahmassebi, F S Haddad and A Robertson (2019) Return to sport after lower limb arthroplasty - why not for all? World J Orthop 10(2): 90-100. https://doi.org/10.5312/wjo.v10.i2.90

22. Kim, M S, I J Koh, S Sohn, J H Jeong and Y In (2019) Unicompartmental knee arthroplasty is superior to high tibial osteotomy in post-operative recovery and participation in recreational and sports activities. Int Orthop 43(11): 2493-2501. https://doi.org/10.1007/s00264-018-4272-5

23. Kleeblad, L J, S M Strickland, B U Nwachukwu, G Kerkhoffs and A D Pearle (2020) Satisfaction with return to sports after unicompartmental knee arthroplasty and what type of sports are patients doing. Knee 27(2): 509-517. https://doi.org/10.1016/j.knee.2019.11.011

24. Lawrence, K W, D A Bloom, V Rajahraman, C Cardillo, R Schwarzkopf, J C Rozell and A Arshi (2024) Return to athletics after total knee arthroplasty: a survey study of 784 recreational athletes across 12 sports. Arch Orthop Trauma Surg 144(9): 4115-4124. 10.1007/s00402-024-05364-9

25. Legnani, C, V Macchi, G M Peretti, G Coccioli, E Borgo and A Ventura (2023) Return to sports and physical activities following mobile-bearing unicompartmental knee replacement. Journal of Biological Regulators and Homeostatic Agents 37(12): 6545-6549. https://doi.org/10.23812/j.biol.regul.homeost.agents.20233712.619

26. Lo Presti, M, G G Costa, S Cialdella, G Agrò, A Grassi, S Caravelli, M Mosca, G M Marcheggiani Muccioli and S Zaffagnini (2019) Return to Sports after Unicompartmental Knee Arthroplasty: Reality or Utopia? A 48-Month Follow-Up Prospective Study. J Knee Surg 32(2): 186-191. https://doi.org/10.1055/s-0038-1635111

27. Lombardi, A V, Jr., K R Berend, C A Walter, J Aziz-Jacobo and N A Cheney (2009) Is recovery faster for mobile-bearing unicompartmental than total knee arthroplasty? Clin Orthop Relat Res 467(6): 1450-1457. 10.1007/s11999-009-0731-z

28. Long, W J, C D Bryce, C S Hollenbeak, R W Benner and W N Scott (2014) Total knee replacement in young, active patients: long-term follow-up and functional outcome: a concise follow-up of a previous report. J Bone Joint Surg Am 96(18): e159. 10.2106/jbjs.M.01259

29. Maugard, L P, C Foissey, C Batailler, S Lustig and E Servien (2025) Return to physical activity after revision total knee arthroplasty: Comparative insights with primary procedure. Knee Surg Sports Traumatol Arthrosc 33(7): 2537-2544. 10.1002/ksa.12638

30. McKay, A K A, T Stellingwerff, E S Smith, D T Martin, I Mujika, V L Goosey-Tolfrey, J Sheppard and L M Burke (2022) Defining Training and Performance Caliber: A Participant Classification Framework. Int J Sports Physiol Perform 17(2): 317-331. https://doi.org/10.1123/ijspp.2021-0451

31. Meena, A, E Abermann, C Hoser, L Farinelli, C Hepperger, A Raj, M K Patralekh and C Fink (2023) No difference in sports participation and patient-reported functional outcomes between total knee arthroplasty and unicompartmental knee arthroplasty at minimum 2-year follow-up in a matched control study. Knee Surg Sports Traumatol Arthrosc 31(8): 3098-3105. https://doi.org/10.1007/s00167-022-07166-1

32. Naylor, J M, N Pocovi, J Descallar and K A Mills (2019) Participation in Regular Physical Activity After Total Knee or Hip Arthroplasty for Osteoarthritis: Prevalence, Associated Factors, and Type. Arthritis Care Res (Hoboken) 71(2): 207-217. https://doi.org/10.1002/acr.23604

33. Peters, M, Y Pronk and J M Brinkman (2024) Eight of ten patients return to daily activities, work, and sports after total knee arthroplasty. World J Orthop 15(7): 608-617. 10.5312/wjo.v15.i7.608

34. Pietschmann, M F, et al. (2013) Sports activities after medial unicompartmental knee arthroplasty Oxford III-what can we expect? Int Orthop 37(1): 31-37. https://doi.org/10.1007/s00264-012-1710-7

35. Pioger, C, J P Bellity, R Simon, O Rouillon, B J Smith and R Nizard (2020) A Playtime and Handicap Analysis of 143 Regular Golfers After Total Knee Arthroplasty at Minimum 2-Year Follow-Up. J Arthroplasty 35(5): 1257-1261. https://doi.org/10.1016/j.arth.2020.01.005

36. Plassard, J, J B Masson, M Malatray, J Swan, F Luceri, J Roger, C Batailler, E Servien and S Lustig (2020) Factors lead to return to sports and recreational activity after total knee replacement - A retrospective study. Sicot j 6: 11. https://doi.org/10.1051/sicotj/2020009

37. Sarrami, M, G Awwad and D Parker (2025) Return to sport after total knee arthroplasty: an Australian perspective. Arch Orthop Trauma Surg 145(1): 236. 10.1007/s00402-025-05843-7

38. Schmidt, A, C Jacquet, C Pioger, S Parratte, J N Argenson and M Ollivier (2023) Retrospective analysis of return to impact sport after medial unicompartmental knee arthroplasty based on a cohort of 92 patients. Orthop Traumatol Surg Res 109(4): 103577. 10.1016/j.otsr.2023.103577

39. Schneider, B L, D I Ling, L J Kleebad, S Strickland and A Pearle (2020) Comparing Return to Sports After Patellofemoral and Knee Arthroplasty in an Age- and Sex-Matched Cohort. Orthop J Sports Med 8(10): 2325967120957425. https://doi.org/10.1177/2325967120957425

40. Streck, L E, C Hanreich, A D Cororaton, C S Boettner and F Boettner (2023) Predictors for activity following total and unicompartmental knee arthroplasty. Arch Orthop Trauma Surg 143(11): 6815-6820. 10.1007/s00402-023-04973-0

41. Taylor, A J, D L Braathen, B E Blackburn, C L Peters, C E Pelt, J M Gililland, M J Archibeck and L A Anderson (2025) Can I Pickle Doc? Return to Pickleball and Other Racket Sports After Total Joint Arthroplasty. J Arthroplasty 40(8): 1979-1986.e1971. 10.1016/j.arth.2025.01.027

42. Tramer, J S, L M Maier, E A Klag, A S Ayoola, M A Charters and W T North (2022) Return to Play and Performance in Golfers After Total Knee Arthroplasty: Does Component Type Matter? Sports Health 14(3): 433-439. https://doi.org/10.1177/19417381211019348

43. Vail, T P, W J Mallon and R A Liebelt (1996) Athletic activities after joint arthroplasty. Sports Medicine and Arthroscopy Review 4(3): 298.

44. Ventura, A, V Macchi, E Borgo and C Legnani (2022) Shift to low-impact sports and recreational activities following total knee replacement. Int J Artif Organs 45(11): 952-956. https://doi.org/10.1177/03913988221119524

45. Walker, T, T Gotterbarm, T Bruckner, C Merle and M R Streit (2015) Return to sports, recreational activity and patient-reported outcomes after lateral unicompartmental knee arthroplasty. Knee Surg Sports Traumatol Arthrosc 23(11): 3281-3287. https://doi.org/10.1007/s00167-014-3111-5

46. Walton, N P, I Jahromi, P L Lewis, P J Dobson, K R Angel and D G Campbell (2006) Patient-perceived outcomes and return to sport and work: TKA versus mini-incision unicompartmental knee arthroplasty. J Knee Surg 19(2): 112-116. https://doi.org/10.1055/s-0030-1248089

47. Yamagishi, T, H Suzuki, M Shizuka and T Kikuchi (2006) Sports activity after total knee arthroplasty, in. R. Johnson, J. Shealy, and T. Yamagishi. Editors. (ed). Skying Trauma and Safety, Journal of ASTM International, 3(8): 35-39.

48. Yim, J H, E K Song, H Y Seo, M S Kim and J K Seon (2013) Comparison of high tibial osteotomy and unicompartmental knee arthroplasty at a minimum follow-up of 3 years. J Arthroplasty 28(2): 243-247. https://doi.org/10.1016/j.arth.2012.06.011

49. Zimmerer, A, L Navas, S Kinkel, S Weiss, M Hauschild, W Miehlke and M Streit (2021) Sports activity and patient-related outcomes after fixed-bearing lateral unicompartmental knee arthroplasty. Knee 28: 64-71. https://doi.org/10.1016/j.knee.2020.11.011
